# Serum BDNF Increase After 9-Month Contemplative Mental Training Is Associated With Decreased Cortisol Secretion and Increased Dentate Gyrus Volume: Evidence From a Randomized Clinical Trial

**DOI:** 10.1016/j.bpsgos.2024.100414

**Published:** 2024-11-10

**Authors:** Lara M.C. Puhlmann, Pascal Vrtička, Roman Linz, Sofie L. Valk, Ioannis Papassotiriou, George P. Chrousos, Veronika Engert, Tania Singer

**Affiliations:** a Research Group Social Stress and Family Health, Max Planck Institute for Human Cognitive and Brain Sciences, Leipzig, Germany; b Leibniz Institute for Resilience Research, Mainz, Germany; c Centre for Brain Science, Department of Psychology, University of Essex, Colchester, United Kingdom; d Otto Hahn Group Cognitive Neurogenetics, Max Planck Institute for Human Cognitive and Brain Sciences, Leipzig, Germany; e Institute of Neuroscience and Medicine (INM-7: Brain & Behaviour), Research Centre Jülich, Jülich, Germany; f Institute of Systems Neuroscience, Medical Faculty, Heinrich-Heine-Universität Düsseldorf, Düsseldorf, Germany; g Department of Clinical Biochemistry, Aghia Sophia Children’s Hospital, Athens, Greece; h First Department of Pediatrics, National and Kapodistrian University of Athens Medical School, Aghia Sophia Children’s Hospital, Athens, Greece; i Institute for Psychosocial Medicine, Psychotherapy and Psychooncology, Jena University Hospital, Friedrich-Schiller-University, Jena, Germany; j German Center for Mental Health, partner site Halle-Jena-Magdeburg, Jena, Germany; k Center for Intervention and Research on adaptive and maladaptive brain Circuits underlying Mental Health, Halle-Jena-Magdeburg, Jena, Germany; l Social Neuroscience Laboratory, Max Planck Society, Berlin, Germany

**Keywords:** BDNF, Cortisol, Hippocampus, Meditation, Mindfulness-based training, Stress reduction

## Abstract

**Background:**

In this study, we investigated whether mindfulness- and meditation-based mental training that improves stress regulation can upregulate BDNF (brain-derived neurotrophic factor), an important promoter of hippocampal neuroplasticity, and examined cortisol reduction as a mediating pathway.

**Methods:**

In a randomized clinical trial, 332 healthy adults were randomly assigned to one of the 3 training cohorts or a passive control cohort. Training participants completed up to three 3-month-long modules targeting attention-based mindfulness, socio-affective skills, or socio-cognitive skills. We examined change in serum BDNF levels after each 3-month training interval; evaluated whether training effects were linked to reduced cortisol release in the long-term, diurnally, and when acutely stress-induced; and explored associations with hippocampal volume changes.

**Results:**

In the combined training cohorts, BDNF increased significantly and cumulatively after 3-, 6-, and 9-month training relative to the pretraining baseline (3 month: *t*_516_ = 3.57 [estimated increase: 1353 pg/mL], 6 month: *t*_516_ = 3.45 [1557 pg/mL], 9 month: *t*_516_ = 3.45 [2276 pg/mL]; all *p*s < .001). After 9 months, training cohort BDNF was not higher than control cohort BDNF, which displayed unexplained variance. However, moderated mediation analysis showed that only training effects, and not control cohort BDNF change, were partially mediated by simultaneously reduced long-term cortisol release (3-month averages) measured in hair (15.1% mediation, *p* = .021). Individually greater BDNF increase after training correlated with more reduced long-term and stress-induced cortisol release. Moreover, greater BDNF increase after 9 months of training correlated with dentate gyrus volume increase (*t*_108_ = 2.09, *p* = .039).

**Conclusions:**

Longitudinal contemplative training may promote a neurobiological pathway from stress reduction to increased BDNF levels to enhanced hippocampal volume. However, single serum BDNF measurements can be unreliable for assessing long-term neurotrophic effects in healthy adults. Future studies should investigate nonspecific BDNF measurement effects before considering application in preventive health care.

## Plain Language Summary

We examined whether meditation-based mental training that reduces psychological stress can increase BDNF (brain-derived neurotrophic factor), a protein that supports brain health. In a randomized trial, 332 adults underwent up to 9 months of training focused on attention-based mindfulness and affective and cognitive skills, or were assigned to a control condition. Results showed significant increases in BDNF over training time, mediated by reduced long-term secretion of the stress hormone cortisol. Increased BDNF was also linked to greater dentate gyrus volume of the hippocampus. However, unexpected BDNF changes in the control condition limit the conclusions that can be drawn. While promising, the findings also highlight the need for more reliable BDNF measures before applying these results to preventive health care.

BDNF (brain-derived neurotrophic factor) is the most frequently expressed neurotrophin in the human brain, where it facilitates neuronal growth, differentiation, and synaptic plasticity throughout neurodevelopment into adulthood (1, 2, 3). While BDNF exerts its neurotrophic effects centrally, human studies typically assay peripheral BDNF levels that correlate highly with central BDNF expression (4, 5, 6). Substantial research has linked lowered peripheral BDNF to decline in brain health and plasticity, in natural aging and Alzheimer’s disease (7, 8, 9), and also in stress-related mood disorders that involve neuronal atrophy (10, 11, 12, 13), such as major depression and bipolar disorder (14, 15, 16, 17, 18).

A causal pathway can be drawn from prolonged psychosocial stress exposure to lowered BDNF levels, consequently impaired neuronal integrity, and finally disorder development. Most prominently, the neurotrophic hypothesis of depression (10,19,20) implicates BDNF as a key mediator in the etiology of stress-related mood disorders. The allostatic load concept (21,22) further describes the cumulative costs of continued stressor adaptation to peripheral systems and the brain (23), where BDNF mediates adaptive plasticity in stress-sensitive regions like the hippocampus, which turn maladaptive under prolonged stress and if BDNF release is inhibited (24). While acute stress increases BDNF levels (25), animal models have confirmed that long-term stress exposure lowers hippocampal BDNF expression (26, 27, 28) [see e.g., (20) for similar patterns in humans].

If stress-related BDNF downregulation leads to neuronal atrophy and disorder, a stress-reducing intervention, in turn, could increase BDNF levels, thus promoting brain health and potentially even counteracting disorder development as a preventive intervention. Contemplative science is an emerging field of research that explores the capacity of meditation and mindfulness- or compassion-based mental training to reduce stress and promote mental and physical health not only in patients but also in the general population (29, 30, 31, 32). Typical contemplative mental training programs, such as the mindfulness-based stress-reduction program (33) or the self-compassion program (34), involve 6- to 8-week-long training in secularized Eastern meditation practices combined with elements from Western cognitive behavioral therapy. Recently, we also developed a 9-month mental training program, the ReSource Project, that compares attention-based mindfulness with socio-affective and socio-cognitive practices in 3 distinct training modules (29,35).

Self-reported stress reduction is among the most frequent health benefits of contemplative training for healthy individuals (36), which has been increasingly corroborated by studies that have shown attenuated endocrine stress responses, measured as lowered levels of the stress hormone cortisol (37), including our own (38, 39, 40). However, it remains unclear whether contemplative training also affects BDNF levels as a more downstream health outcome, especially in the general population. Current research has provided only limited evidence for BDNF increase after 6- to 12-week-long interventions of varying quality and predominantly in patients (41, 42, 43).

Endocrine stress reduction has been proposed as a pathway through which contemplative interventions may increase BDNF levels and promote brain health (44,45). A well-functioning hypothalamic-pituitary-adrenal (HPA) axis, which is one of our 2 main endocrine stress systems, facilitates adaptation to environmental demands by secreting glucocorticoids (cortisol and cortisone) in a dynamic diurnal rhythm and in response to acute stressors (22). However, prolonged stress leads to maladaptive HPA axis activity, typically characterized by flattened diurnal cortisol curves (46), inability to downregulate acute responses (47,48), and overall greater cortisol release. These changes contribute to downstream negative health outcomes (21,22), presumably including BDNF downregulation (10). A direct antagonistic cortisol-BDNF relationship has been identified during chronic stress (10,26), in acute stress induction (25), and in opposing long-term effects of glucocorticoid and BDNF signaling on stress-sensitive regions like the hippocampus (48, 49, 50, 51).

Here, we examined whether the 9-month long ReSource mental training program of attention-based mindfulness and cognitive-affective skills increases circulating BDNF levels of healthy adults and whether the hypothesized training effect is mediated by reduced cortisol release. Our previous work in the ReSource Project identified 3 potential mediators: 1) lowered cortisol awakening response (CAR) after socio-affective training (40), 2) reduced acute stress-induced cortisol increase after socio-affective and socio-cognitive training (39), and 3) lowered long-term average cortisol release after 6 to 9 months training, irrespective of content (measured as hair cortisol concentration [HCC] and hair cortisone concentration [HEC]) (38). These findings demonstrate the in-principle stress-reducing properties of all ReSource modules. Consequently, we predicted that all 3 modules could increase BDNF levels. Next to mediation of the group-level effects of training, we examined on the participant level whether individually greater BDNF increase after training covaried with greater reduction in diurnal, acutely stress-induced, and long-term cortisol secretion.

To explore neurobiological implications of the hypothesized BDNF increase, we also tested its relationship to hippocampal volume and specifically the dentate gyrus subfield. The hippocampus is sensitive to the damaging effects of prolonged glucocorticoid signaling (10,19) and displays remarkable lifetime structural plasticity, which is in large part mediated by BDNF action (2). Among the cytoarchitecturally distinct subfields of the hippocampus (52), the dentate gyrus appears to be particularly susceptible to the effects of BDNF (53) and was thus our main target next to total hippocampal volume, consistent with our hypotheses in a previous preregistered study (54). Specifically, we expected that training-related BDNF increase might be associated with, or lead to, greater dentate gyrus and total hippocampal volume.

## Methods and Materials

### Participants

**Figure 1:**
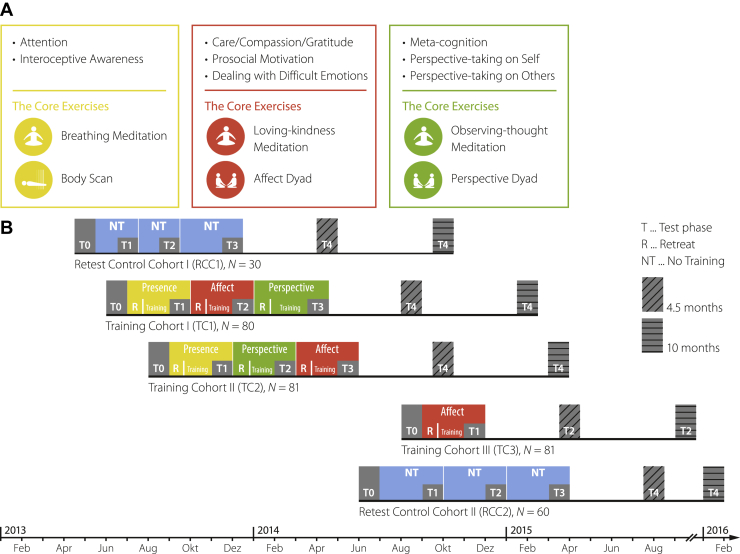
Design and timeline of the ReSource Project. **(A)** Key concepts and core exercises taught as part of the 3 training modules; presence (yellow, left panel), affect (red, middle panel), and perspective (green, right panel) (112). The presence module aimed to cultivate present moment–focused attention and interoception with 2 widely used meditation techniques, body scan and breathing meditation, as daily core exercises. The affect module was designed to cultivate socio-affective qualities, such as compassion, gratitude, and nonjudgmental acceptance of difficult emotions, using loving-kindness meditation and affect dyad as core exercises. The affect dyad is a partner-based exercise specifically designed for the ReSource Project that aims to cultivate socio-affective abilities with a weekly changing partner in 10-minute dyadic practices (113). The perspective module targeted socio-cognitive and metacognitive analytic abilities; its daily core practices included observing-thoughts meditation (112) and the perspective dyad, another type of intersubjective daily practice that is similar in structure to the affect dyad but that teaches cognitive perspective taking (113). **(B)** Timeline of the ReSource Project. Two separate training chohorts (TC1 and TC2) completed the 3 modules in different orders; a third cohort (TC3) completed only the affect module to isolate the specific effects of this module. The training time was 13 weeks per module, totaling 39 weeks for TC1 and TC2. The retest control cohort (RCC) underwent all testing but no training (blue squares). Retest control participants were recruited in 2 cohorts for logistic reasons but were analyzed jointly. Blood samples for the assessment of serum BDNF (brain-derived neurotrophic factor), magnetic resonance imaging scans for brain morphology, hair strands for calculation of hair cortisol concentration and hair cortisone concentration, and saliva samples for estimating diurnal cortisol secretory patterns were each acquired at study baseline (T0) and after 3, 6, and 9 months (T1–T3). Stress-induced cortisol was only sampled once per participant at T0, T1, or T2, each time from a subset of participants from each cohort, due to concerns about retest effects given the deceptive nature of the stress induction [also see (39)]. Follow-up assessments (T4) were conducted with a subset of participants, but these measures are not part of the current investigation. Further details on the study design have been reported elsewhere (35). Figure reproduced and adapted with permission from (35,114).

As part of the ReSource Project (35), meditation-naïve healthy adults were recruited from the general public and screened for Axis I and Axis II disorders by a trained clinical psychologist (55,56) (Supplementary Methods). An initial sample of *N* = 332 participants (175 women; mean age [SD]: 40.5 [9.3] years) was randomly distributed across a passive retest control cohort (RCC) (*n* = 90) and 3 training cohorts (TCs) (TC1, *n* = 80; TC2, *n* = 81; TC3, *n* = 81) (Figure 1B) using bootstrapping without replacement. Participants gave written informed consent, could withdraw from the study at any time, and were financially compensated for their participation. The study was conducted in compliance with the Declaration of Helsinki and approved by the research ethics committees of the University of Leipzig (Ethic No. 376/12-ff) and Humboldt University in Berlin (Ethic Nos. 2013-20, 2013-29, 2014-10), Germany [see (57) for additional, extensive detail on participants and recruitment].

### Training Protocol

The ReSource Project compared the effects of 3 distinct types of mental training, specifically attention-, socio-affective-, or socio-cognitive-based techniques. For this purpose, the training program was parceled into 3 separate modules termed presence, affect, and perspective, each cultivating distinct contemplative capacities (Figure 1A) (35). Each module was trained for 3 months and began with a 3-day silent retreat. For the subsequent 13-week training period, participants attended weekly 2-hour group sessions with expert teachers and performed 2 core practices at home for approximately 30 minutes/day, 5 days/week, using a tailor-made app and online platform.

### Study Design and Sampling

The ReSource study followed a mixed design in which most training cohort participants received all types of training in partially counterbalanced order (Figure 1B). All biosamples except acute stress-induced cortisol release were repeatedly acquired at study baseline (T0) and after 3, 6, and 9 months (T1–T3), corresponding to completion of each 3-month training module (35). RCC participants underwent all testing but no training, providing an estimate of retest effects relevant to measures of the ReSource Project that were not analyzed here, such as questionnaires.

### Measures

The examined indices of cortisol release and hippocampal morphology are summarized in Table 1. Detailed descriptions of the assessment protocols for these measures are provided in Supplementary Methods and in previous publications (38,39,54,58).

**Table 1:** Indices of HPA Axis Activity and Hippocampal Morphology and Their Computation

Measure (Abbreviation)	Indicator of	Modality	Calculation
Long-Term Average Cortisol Release			
Hair Cortisol Concentration (HCC)	Long-term (3 mo) HPA axis activity	Hair	Total cortisol accumulation in 3-cm hair segments (reflecting average cortisol exposure over 3 mo)
Hair Cortisone Concentration (HEC)	Long-term (3 mo) HPA axis activity	Hair	Total cortisone accumulation in 3-cm hair segments (reflecting average cortisone exposure over 3 mo)

Stress-Induced Cortisol Release			
Baseline-to-Peak Cortisol Increase (C_inc_)	HPA axis reactivity	Saliva	Cortisol level at average peak time (20 min) corrected for cortisol at pre-TSST baseline (−20 min) via residualization
Minimum Cortisol Level (C_min_)	HPA axis recovery	Saliva	Individually lowest cortisol value throughout the stress induction
Maximum Cortisol Level (C_max_)	Maximum HPA axis activation	Saliva	Individually highest individual cortisol value throughout the stress induction
Absolute Cortisol Difference (C_maxmin_)	Alternative index of HPA axis reactivity	Saliva	Difference score between individually lowest (C_min_) and highest (C_max_) cortisol values throughout the stress induction

Diurnal Cortisol Release			
Cortisol Awakening Response (CAR)	Diurnal preparatory HPA axis activation	Saliva	Difference score between first measurement to the 30-min postawakening sample; corrected for time of awakening and cortisol levels at awakening
Diurnal Cortisol Slope (Cslope)	Overall dynamics in diurnal HPA axis activation	Saliva	Difference score between first and final cortisol sample of the day at 600 minutes after awakening; corrected for time of awakening and cortisol levels at awakening
Total Diurnal Cortisol Output (CAUC)	Total diurnal HPA axis activity	Saliva	Cortisol AUC with respect to ground (77) using baseline, 240-, 360-, 480-, and 600-min postawakening cortisol values; corrected for time of awakening and cortisol levels at awakening

Hippocampal Morphology			
Dentate Gyrus Volume (DGV)	Dentate gyrus volume	MRI	Left and right DG/CA4 subfields
Total Hippocampal Volume (HCV)	Hippocampal volume	MRI	Sum of the left and right DG/CA4, CA, and SUB subfields

Indices were derived from previous studies of training effects on long-term average cortisol release (38), stress-induced cortisol release (39), diurnal cortisol release (40), and hippocampal volume (104) [also see (54)]. To examine potentially differential associations of stress-induced reactivity and recovery with BDNF (brain-derived neurotrophic factor), we also examined 3 relatively unadulterated 1-index measures of acute cortisol dynamics, the minimal (C_min_), maximal (C_max_), and change between minimal and maximal cortisol concentration (C_maxmin_) throughout the stress-induction period. C_maxmin_ and C_min_ were proposed as optimal indices of reactivity and recovery in a data-driven analysis (65) and used in our previous work (47); C_max_ was also included due to the marked numeric change of peak cortisol levels following training (39). All 3 indices of diurnal cortisol release were corrected for time of awakening and awakening cortisol levels (40,115).

AUC, area under the curve; CAUC, cortisol area under the curve; C_inc_, cortisol increase; DG, dentate gyrus; HPA, hypothalamic-pituitary-adrenal; MRI, magnetic resonance imaging; SUB, subiculum; TSST, Trier Social Stress Test.

#### Brain-Derived Neurotrophic Factor

BDNF concentration was added as an outcome to the ReSource Project subsequent to trial registration in 2013 given growing interest in BDNF interaction with training-induced stress reduction and its potentially mediating role in neurocognitive changes (44). Peripheral BDNF levels were determined in serum, which is a more reliable marker than plasma BDNF (59). To control diurnal fluctuations, each participant's blood samples were taken at the same time of day throughout the study (mean deviation in sampling time [SD]: −0.087 [2.30] hours). Blood was allowed to clot for 30 to 45 minutes and subsequently centrifuged at 3500 rpm for 15 minutes, after which serum was frozen at −80 °C until assay with a quantitative sandwich enzyme immunoassay technique (R&D Systems, Inc.). The intra- and interassay coefficients of variation of <7% were determined by duplicate analysis of >6% of randomly selected samples. We examined baseline (T0) BDNF data in a previous study (54).

#### Cortisol Measures: Long-Term Average Cortisol Release

HCC and HEC indicate systemic glucocorticoid exposure and chronic stress (60,61). Hormone concentrations in proximal 3-cm segments of hair were analyzed to assess their average accumulation over 3 months (61,62), corresponding to the 3-month training intervals. Cortisone is the inactive metabolite and precursor molecule to cortisol and yields a complementary estimate of cortisol exposure (63). To avoid straining participants through excessive testing, hair sampling was presented as an optional rather than a core testing procedure, which led to lower adherence rates (see Table S1) (38).

#### Cortisol Measures: Stress-Induced Cortisol Release

Saliva samples were collected during the Trier Social Stress Test (64) for psychosocial stress induction, at −55, 10, 20, 30, 40, and 55 minutes relative to stressor onset. We computed 4 indices of stress-induced cortisol release: Stress reactivity was measured as baseline-to-peak cortisol increase, which we previously found lowered after training (39). Another hallmark of healthy HPA axis functioning is a timely stress recovery (22,48), which we recently found was differentially related to health outcomes compared to reactivity (25,47). To disentangle increase and recovery, we examined the minimal (C_min_), maximal (C_max_), and change between minimal and maximal cortisol concentration (C_maxmin_) during the stress testing session (see Table 1) (47,65).

#### Cortisol Measures: Diurnal Cortisol Release

On 2 days per testing time point (T0–T3), saliva was sampled upon awakening and 30, 60, 240, 360, 480, and 600 minutes thereafter. We computed 3 diurnal cortisol indices [see (40,66,67)] (Table 1): the CAR, which represents the physiological enhancement that is activated to deal with the anticipated daily demands of the upcoming day (68, 69, 70); the diurnal cortisol slope (Cslope), which describes cortisol decline over the day [a steeper negative slope is considered an indicator of healthy HPA axis functioning (71)]; and cortisol area under the curve (72), which represents total diurnal cortisol output and exposure (71).

#### Hippocampal Volume

All magnetic resonance imaging data for the calculation of dentate gyrus volume (DGV) and total hippocampal volume (HCV) were acquired using the same imaging hardware and console software (Syngo B17). Hippocampal volumes were estimated based on T1-weighted data linearly registered to MNI152, implicitly controlling for intracranial volume. CA1–3, CA4/DG, and subiculum hippocampal subfields were segmented using a validated patch-based algorithm in every participant (73), following our previous approach (54). This algorithm has high accuracy for segmenting hippocampal subfields in T1 images with similar resolution (73), which we also controlled through intensive manual checks by 2 independent raters, RL and LMCP (54). To facilitate the comparability of our results, we also estimated total hippocampal and subfield volumes using a FreeSurfer tool for automated segmentation (74). Findings were consistent across both segmentation approaches.

### Statistical Analysis

#### Training Effects on BDNF

We first evaluated whether BDNF levels differed as a function of training routine over time by testing the interaction between cohort assignment (TC1–TC3, RCC) and time point (T0–T3) in a multilevel model (termed “cohortwise model”). Consistent with our previous work (54), age and sex were predefined as the only covariates (8,75, 76, 77) (see Supplementary Methods for model equation). To boost analysis power and given overall cumulative BDNF change (see Results), we further modeled training effects in the combined cohorts as the main effect of training duration (termed “combined model”). Follow-up contrasts comparing the effects of individual training modules and time points were subsequently conducted within the multilevel model framework and did not require correction for multiple comparisons. We contrasted BDNF estimates at each time point post baseline (T1, T2, T3) with 1) the matched time point in the RCC (between-cohort contrasts) and 2) the pretraining (baseline) BDNF levels of the respective cohort (within-cohort contrasts). Analyses were conducted in R (version 4.2.0) (78), models were fit using the package lme4 (79), contrast calculated using the function “emmeans” (80), and effect sizes calculated as omega squared (ω^2^) (81).

#### Mediation Analyses

We tested for the presence of indirect training effects on BDNF via reduced cortisol exposure through 1) mediation analysis in the combined training cohorts (contemporaneous and lagged) and 2) moderated mediation in the training cohorts compared with the control cohort. Mediation analyses were calculated using the function “mediate” of the R package “mediation” (82) and thus conducted in the framework of causal mediation. This approach follows a counterfactual mediation framework and extends traditional mediation approaches by enhancing the accuracy of effect decomposition into direct and indirect effects, among other improvements (83).

#### Change Covariance

We explored associations between participant-level BDNF change (difference scores, ΔBDNF) and concurrent change in cortisol and hippocampal volume indices (Table 1). To this end, individual difference scores were calculated from the pretraining baseline (T0) to maximum training duration (9 months/T3). We targeted maximum training duration to account for cumulative BDNF change (see Results), the slow-changing nature of long-term cortisol (38) and presumably HCV, and to avoid multiple testing. Therefore, these analyses did not include TC3 participants, who trained for 3 months only (see Figure 1).

## Results

In total, 1042 BDNF observations were available from 322 participants (see Table 2 for sample characteristics, Table S1 for available *n* per measure, and Supplementary Results A and Figure S1 for baseline associations and comparisons). The short 3-month cohort TC3 had lower values than all other study cohorts (omnibus test: *F*_2,310_ = 3.84, *p* = .010) (Table S3). Relevant demographic variables, including age, sex, IQ, and self-reported traits such as subjective stress levels, were explicitly counterbalanced across cohorts (35). Nonetheless, to account for baseline differences, cohort assignment was included as a covariate in all subsequent analyses.

**Table 2:** Descriptive Statistics for Baseline Demographic Variables Separately by Cohort

Measure	RCC, *n* = 80	TC1, *n* = 75	TC2, *n* = 80	TC3, *n* = 80
Age, Years	39.61 (9.30)	40.92 (9.02)	41.19 (9.86)	40.65 (8.92)
Sex, Male	33 (41.2%)	32 (42.7%)	32 (40.0%)	32 (40.0%)
Hormonal Status				
Male	33 (41.2%)	32 (44.4%)	32 (40.5%)	32 (41.6%)
No cycle	13 (16.2%)	10 (13.9%)	14 (17.7%)	9 (11.7%)
Oral contraceptive	9 (11.2%)	6 (8.3%)	9 (11.4%)	11 (14.3%)
Natural cycle	25 (31.2%)	24 (33.3%)	24 (30.4%)	25 (32.5%)
Smoker	67 (90.5%)	67 (89.3%)	66 (82.5%)	58 (89.2%)
BDNF, pg/ml	24,916 (5360)	25,881 (5743)	25,524 (6542)	23,020 (5779)
Long-Term Average Cortisol Release				
HCC, pg/mg	6.62 (7.35)	6.67 (7.48)	8.12 (6.61)	8.90 (13.53)
HEC, pg/mg	10.76 (9.80)	10.33 (7.16)	13.38 (9.16)	12.80 (9.10)
Stress-Induced Cortisol Release				
C_inc_	0.06 (0.69)	0.40 (0.62)	0.09 (0.49)	0.03 (0.39)
C_max_, log nmol/L	8.65 (5.24)	13.69 (10.16)	8.93 (4.73)	8.23 (4.82)
C_min_, log nmol/L	2.88 (1.84)	4.10 (2.41)	2.96 (1.13)	3.41 (2.12)
C_maxmin_, log nmol/L	5.77 (4.37)	9.59 (9.15)	5.96 (3.88)	4.82 (3.09)
Diurnal Cortisol Release				
CAR	0.45 (0.57)	0.46 (0.48)	0.48 (0.53)	0.49 (0.55)
Cslope	−1.33 (0.72)	−1.27 (0.49)	−1.46 (0.59)	−1.37 (0.66)
CAUC	750.67 (247.89)	732.32 (168.75)	751.23 (201.94)	705.42 (258)
Hippocampal Volume				
DGV, mm^3^	1270 (157)	1267 (166)	1287 (155)	1282 (124)
HCV, mm^3^	12,352 (959)	12,551 (1138)	12,368 (934)	12,511 (788)

Vaues are presented as mean (SD) or *n* (%). The descriptive statistics shown here are for the sample of participants who provided baseline serum BDNF data at baseline (in total, 1042 BDNF observations were available from 322 participants, with 7 participants missing BDNF data at baseline) (see Table S1 for participant numbers per time point and cohort across all individual difference variables of interest). Baseline descriptives are shown as raw data, with the exception of cortisol reactivity (C_inc_) and diurnal cortisol indices, which were computationally derived [see (40)] (see the Supplement for a depiction of raw diurnal cortisol data).

Previous studies of the ReSource Project reported main training effects on the assessed cortisol measures (38, 39, 40) or the relationship between BDNF and cortisol stress reactivity and recovery (25). For the current work, we exclusively focused on basal BDNF levels and cortisol samples available in the same participants. Thus, differences in the respective study samples exist due to different overlaps in missing data points for basal BDNF and the respective cortisol measures.

BDNF, brain-derived neurotrophic factor; CAR, cortisol awakening response; CAUC, cortisol area under the curve; C_inc_, cortisol increase; DGV, dentate gyrus volume; HCC, hair cortisol concentration; HCV, total hippocampal volume; HEC, hair cortisone concentration; RCC, retest control cohort; TC, training cohort.

### Training Effects on BDNF: Cohortwise Model

**Figure 2:**
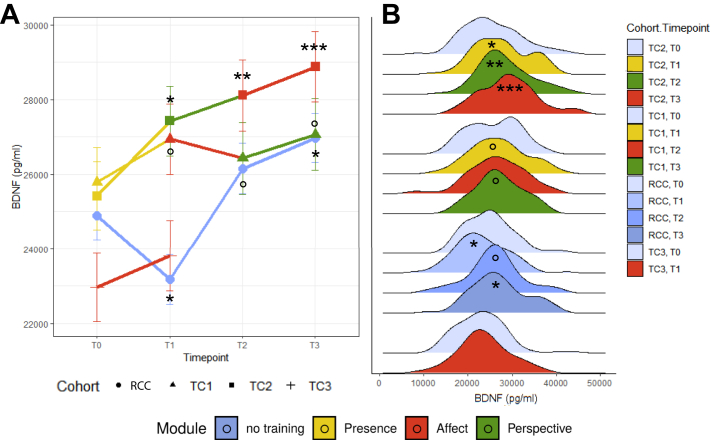
BDNF (brain-derived neurotrophic factor) change by cohort and time point. **(A)** Estimated average BDNF levels per time point and cohort (training cohort [TC] 1, TC2, TC3, retest control cohort [RCC]). In the framework of a multilevel model fit across all participants and data, estimated BDNF levels after each training module were compared within participants to BDNF levels at the pretraining baseline (T0). Within-subject contrasts provide particularly sensitive assessments of change while controlling for implicit covariates, such as individual differences in biology and general propensity to hormone release. In other studies of the ReSource Project, between-subject comparisons to RCC (blue) instead served as the main points of reference for outcome measures with potential retest effects, such as tasks-based assessments, which were not part of the current investigation. **(B)** Density distributions of raw BDNF data per time point. Asterisks indicate significant differences relative to the respective cohort baseline. ° marginal at .1 > *p* > .05; ∗ significant at *p* < .05, ∗∗ significant at *p* < .01, ∗∗∗ significant at *p* < .001.

The cohortwise multilevel model indicated that, as hypothesized, BDNF levels developed differently over time depending on cohort assignment (significant cohort × time interaction: χ^2^_7_ = 28.28, *p* < .001, ω^2^ = 0.040, 1042 observations) (see Figure 2 and Table S2 for raw BDNF data). Model estimates identified notable BDNF change in the training cohorts and, unexpectedly, also in the RCC.

#### Between-Cohort Contrasts

At T1, BDNF levels in both TC1 and TC2 were significantly higher than in the RCC (T1, TC1: *t*_658_ = 3.99, *p* < .001; T1, TC2: *t*_666_ = 4.52, *p* < .001), which was driven by a combination of BDNF increase in TC1 and TC2 and strong simultaneous BDNF decrease in the RCC. At T2 and T3, BDNF levels in TC2 were significantly higher than in the RCC (T2: *t*_697_ = 2.04, *p* = .042; T2: *t*_676_ = 2.01, *p* = .045) (see Table S4 for all contrasts).

#### Within-Cohort Contrasts

In TC2, BDNF levels increased cumulatively at 3 to 9 months of training relative to the pretraining baseline (T1: *t*_729_ = 3.05, *p* = .002; T2: *t*_734_ = −4.02, *p* < .001; T3: *t*_733_ = 5.18, *p* < .001, maximum estimated increase at T3: 3456 pg/mL). BDNF levels in TC1 were marginally increased at T1 (*t*_722_ = −1.73, *p* = .084) and T3 (*t*_729_ = −1.86, *p* = .063, maximum estimated increase at T3: 1280 pg/mL) relative to baseline. Density plots indicated some unusually low BDNF values at T2 (Figure 2, right), which may have contributed to the nonsignificant contrast at that time point. TC3 showed a nonsignificant increase between T0 and T1 (*t*_737_ = 1.25, *p* = .210, estimated increase: 840 pg/mL).

The overall pattern suggests that BDNF in the training cohorts changed cumulatively as a function of training duration, with some differences between cohorts but no specific effect of training content (see Figure 2 and Table S5).

### Analyses of Potential Confounding Factors in the RCC

Despite not undergoing any training, BDNF levels in the control cohort (RCC) changed significantly over the 9 months of observation, with a drop at T1 and a subsequent increase (Figure 2 and Table S4). We do not have a fully compelling explanation for these fluctuations. Systematic retest effects are not expected for physiological measurements such as BDNF. In longitudinal studies, BDNF measurements can be confounded by seasonal changes, in particular ambient sunlight (84). Here, BDNF was positively correlated with average hours of sunlight in the month preceding sampling, but controlling for sunlight or other seasonal variables neither increased the explained variance nor altered the overall pattern of results in the main model of training effects (Supplementary Results B, Figure S2, and Table S6).

Noisy measurements and insufficient stability can also contribute to inconsistent measurements. Intraclass correlation coefficients (ICCs) of repeated BDNF measurements, an indicator of test-retest reliability, were lowest in the RCC (ICC = 0.436), followed by TC1 (ICC = 0.483), TC2 (ICC = 0.580), and TC3 (ICC = 0.636). Thus, the RCC showed the greatest proportion of within-subject compared with between-subject variance in BDNF, supporting the hypothesis that BDNF measures in this cohort were relatively less reliable.

### Training Effects on BDNF: Combined Model

**Figure 3:**
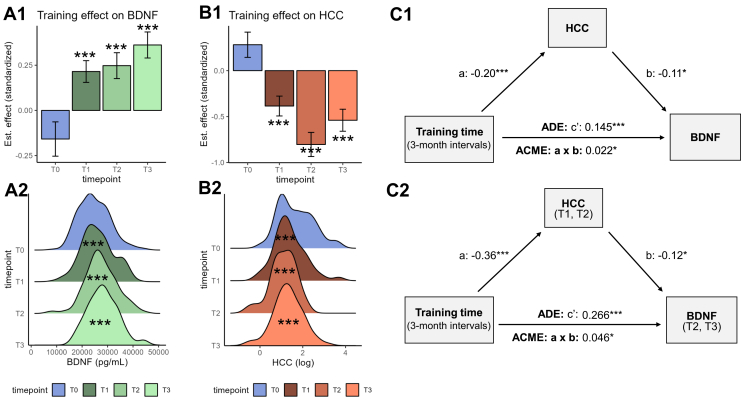
Combined training effects and mediation of training-related BDNF (brain-derived neurotrophic factor) increase via hair cortisol concentration (HCC) reduction. **(A1, B1)** Estimated BDNF levels **(A1)** and HCC **(B1)** of the combined training cohorts at time points T0 to T3, derived from multilevel model fits. Time point effect estimates are centered on training cohort 1, resulting in below/above zero estimates at T0 (see Table S7 for detailed effect estimates). Detailed training effects on HCC have been reported previously (38). **(A2, B2)** Distribution of raw BDNF and log-transformed HCC data per time point. **(C1, C2)** Estimated mediation components (C1: concurrent, C2: lagged) in stepwise notation (86). Effect estimates were derived using quasi-Bayesian Monte Carlo simulation with 10,000 runs and standard uncertainty estimates. Path a: effect of independent variable training (linear effect of posttraining time points) on mediator HCC; path b: association between mediator HCC and outcome variable BDNF, estimated across all time points of measurement; path c’: direct effect of training on BDNF (average direct effect [ADE] in causal mediation notation); path a × b**:** indirect effect of training via HCC reduction (average causal mediation effect [ACME] in causal mediation notation), representing a proportional mediation of 15.1% of the total effect. All effect estimates are fully standardized. ∗ significant at *p* < .05, ∗∗∗ significant at *p* < .001.

To avoid confounding training effect estimates with the unexplained and unsystematic BDNF change in the RCC and given the overall cumulative BDNF increase, we implemented pre–post intervention comparisons in the combined training cohorts as the most powerful test of training effects on BDNF. This combined model showed a main effect of posttraining time point on BDNF levels (χ^2^_3_ = 28.11, *p* < .001, 745 observations from 239 TC participants), with significant and apparently cumulative BDNF increase after 3, 6, and 9 months of training, always compared with the pretraining baseline (T1: *t*_516_ = 3.57, estimated increase [standardized effect in parentheses]: 1353 pg/mL (0.22), *p* < .001; T2: *t*_516_ = 3.45, estimated: 1557 pg/mL (0.25), *p* < .001; T3: *t*_516_ = 5.02, estimated: 2276 pg/mL (0.36), *p* < .001) (see Figure 3A1 and Table S7). We also modeled training duration with a continuous variable posttraining time point to test for a linear effect of training, which was supported (χ^2^_1_ = 24.97, *p* < .001, ω^2^ = 0.049, 745 observations, estimated BNDF increase: 737 pg/mL (0.12) per 3-month training interval).

### Mediation via Cortisol Reduction

**Figure 4:**
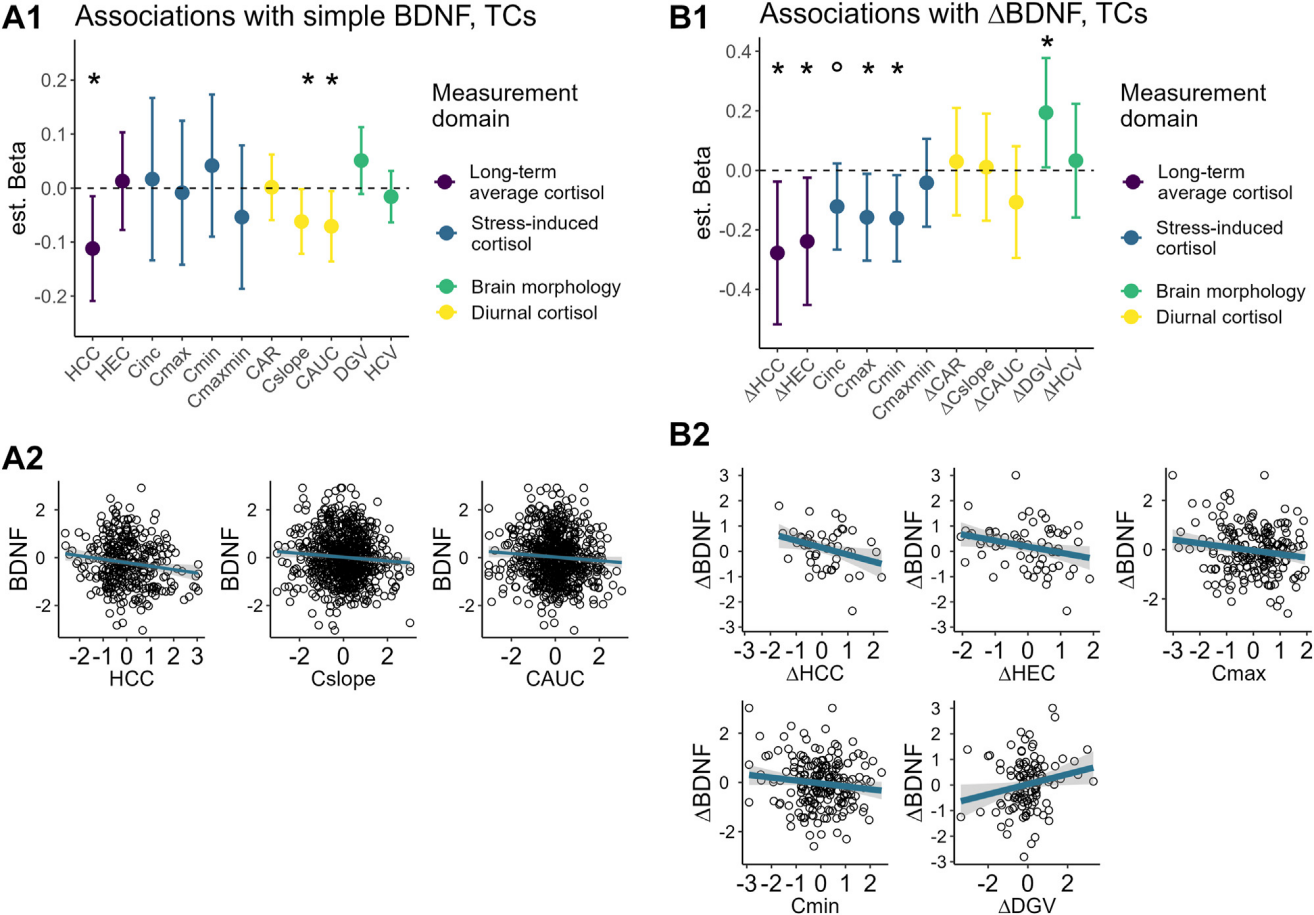
Associations of cortisol and hippocampal volume indices with **(A1, A2)** simple endogenous BDNF (brain-derived neurotrophic factor) levels and **(B1, B2)** individual BDNF change (difference scores, ΔBDNF) in the training cohorts (TCs). **(A1)** Estimated beta values of simple score associations derived from multilevel models fit over data from all time points and training participants. Before testing change score associations, we first examined associations between simple endogenous BDNF levels and simple cortisol, dentate gyrus volume (DGV), and total hippocampal volume (HCV) scores. Besides hair cortisol concentration (HCC), the diurnal cortisol slope (Cslope) and cortisol area under the curve (CAUC) were significantly negatively associated with participants’ simple endogenous BDNF levels (see Table S8)—2 additional indices of overall diurnal cortisol secretion, which were however not reduced by the training intervention (40) and thus did not qualify for mediation analysis. **(A2)** Scatterplots and estimated regression lines for significant simple score associations. **(B1)** Estimated beta values of associations between changes scores from the pretraining baseline (T0) to the maximum training duration (T3), derived from linear models with the combined 9-month training cohorts, TC1 and TC2 (see also Table S10). For the cross-sectionally sampled stress-induced hypothalamic-pituitary-adrenal axis activity, associations between ΔBDNF and simple cortisol indices (C_inc_, C_max_, C_min_, C_maxmin_) were evaluated in all TCs (TC1–TC3). **(B2)** Scatterplots of significant change score associations. All beta estimates are fully standardized. Associations were modeled as BDNF by cortisol indices and HCV/DGV by BDNF, controlling for age, sex, training cohort membership, and time point, respectively. ° marginal at .1 > *p* > .05; ∗ significant at *p* < .05. CAR, cortisol awakening response; C_inc_, cortisol increase; HEC, hair cortisone concentration.

We tested the mediation of group-level BDNF increase in the above combined cohort model of linear training effects (training duration), as sample size requirements are generally large for mediations (85), and to avoid multiple comparisons. Among the 4 previously identified potential cortisol mediators (38, 39, 40), only HCC was associated with BDNF levels (Figure 4A1, A2) and thus qualified for mediation. Figure 3B1 shows the estimated HCC reduction after 3 to 9 months of training.

#### Mediation in the Training Cohorts

Contemporaneous mediation analysis revealed a significant indirect effect of training duration on BDNF via concurrently reduced HCC (15.1% mediation; estimated average causal mediation effect [ACME]: 0.022 [95% CI, 0.003 to 0.05], *p* = .021, *n* = 338 observations from *n* = 149 participants) (see Figure 3C1). Supplementary analyses of training time point as a categorical variable (T0–T3) confirmed that BDNF increase at each posttraining time point was mediated by HCC reduction (ACMEs, 0.043–0.090, 11.1%–32.2% mediation; all *p*s ≤ .25) (see Figure S3 and Table S9). Results are statistically equivalent to the more traditional product method for mediation (82,86).

Lagged mediation analysis further supported an indirect effect of training duration on BDNF at T2 and T3 via HCC reduction at the preceding time points (T1 and T2, respectively) (14.4% mediation; ACME: 0.046 [95% CI, 0.003 to 0.10], *p* = .037, 233 observations from 134 participants) (see Figure 3C2).

#### Moderated Mediation

Moderated mediation analysis confirmed that training assignment moderated the mediation of the linear effect of time point on BDNF via concurrently reduced HCC in that mediation was significant in the training cohorts (ACME: 0.020 [95% CI, 0.001 to 0.04], *p* = .036, 11.6% of total effect) but not in the control cohort (ACME: 0.007 [95% CI, −0.01 to 0.03], *p* > .35, 3.6%) (82).

### BDNF Change Covariance

In the combined training cohorts, ΔBDNF correlated inversely with ΔHCC and ΔHEC after 9 months and with stress-induced cortisol release cross-sectionally (Figure 4B1, B2 and Table S10). Moreover, ΔBDNF after 9 months of training was positively associated with ΔDGV (*t*_108_ = 2.09, *p* = .039) (for completeness, Figure S5 shows ΔBDNF associations with ΔHCV, ΔDGV, and change in the complementary subfields subiculum and cornu ammonis, per hemisphere). By comparison, in the RCC, only an association between ΔBDNF and ΔHCC could be identified (Figure S6 and Table S11).

## Discussion

Downregulation of BDNF has been linked to neuronal atrophy, particularly in the hippocampus, and the subsequent development of stress-related mood disorders (10,21,50). In the ReSource Project (35), we examined whether contemplative mental training of attention-based mindfulness and socio-affective and socio-cognitive skills increases serum BDNF levels of healthy adults. We identified an overall cumulative BDNF increase over 3 to 9 months of training. After 9 months, BDNF in the training cohorts was surprisingly not higher than BDNF in the RCC, which followed an unexplained change pattern. Mechanistically, however, only BDNF increase in the training cohorts was mediated by reduced long-term cortisol exposure. On the participant level, a greater training-related BDNF increase was also associated with greater reductions in most indices of long-term and stress-induced cortisol, while in the RCC, it was only associated with long-term cortisol. Examining hippocampal morphology, a greater BDNF increase after 9-month training was associated with individually greater DGV increase.

The identified main effect of mental training extends a relatively small body of work investigating peripheral BDNF levels after mindfulness-based training, which is often confounded by physical activity and mostly focused on patients (87). A recent meta-analysis of 11 controlled trials summarizes the first evidence for BDNF increases following 6- to 12-week-long mindfulness-based interventions with and without physical activity (41). However, among the included studies, only one relatively short meditation-based mindfulness-based intervention was conducted with healthy adults, who were of older age (mean age 72.9 years). A concurrently published review highlights that the existing evidence for BDNF increase after mindfulness-based interventions is limited and preliminary and calls for more well-designed studies (42) because past trials varied in quality and had limited durations of typically 6 to 8 weeks (41,42). The current intensive longitudinal trial provides the first evidence for long-term endogenous BDNF increase in healthy middle-age adults, accumulated over 9 months of training. BDNF increased after instances of all training modules, with cumulative rather than differential effects. While we observed module-specific effects for many other outcomes of the ReSource Project (29), some skills, including interoceptive body awareness (88) and long-term stress regulation (38), appear to improve after all modules—if given sufficient time.

Our results highlight cortisol release as one pathway through which contemplative training may increase BDNF levels (44,89). Mediation analyses supported the hypothesis that the group-level training effects on BDNF manifested themselves partly indirectly via reduced long-term cortisol exposure measured in hair (HCC) at the concurrent or preceding time point. We found no evidence for mediation via more dynamic aspects of HPA axis activity, namely cortisol increase during stress induction or the diurnal CAR. The specific mediation via long-term cortisol is consistent with the particularly well-supported antagonism between chronic stress and both BDNF action and neuronal integrity (90,91): While acute stress stimulates central and peripheral BDNF levels (25,92), and glucocorticoid and BDNF signaling can interact permissively to facilitate neuroplasticity (92, 93, 94), chronic stress and consequent excess glucocorticoid exposure have consistently been found to downregulate BDNF and impair neuronal integrity (26,28,50). This mechanism may also explain stress- and glucocorticoid-related hippocampal atrophy and related disorder development (50,95).

We previously found that HCC in the RCC remained stable over the 9 months of observation (38). Here, moderated mediation analysis confirmed that BDNF increase in the training cohorts, but not in the RCC, was mediated by concurrent HCC reduction. Relatedly, examining participant-level change scores, training participants who showed greater BDNF increase also displayed stronger reduction in most indices of long-term and stress-induced cortisol release (Figure 4B1 and Table S10). In contrast, individual BDNF change in the control cohort was mostly not associated with simultaneous cortisol reduction, except for HCC (Figure S6 and Table S11). These results embed training-related BDNF increase in broader changes in stress-physiology, thus corroborating its physiological reliability, while RCC BDNF change remains unexplained. Change in seasonal variables like sunlight exposure did not account for RCC BDNF fluctuation. Thus, we suggest that the unexplained change may be a symptom of methodological errors or confounds, considering also that test-retest stability was lowest in this cohort.

To our knowledge, only 2 smaller studies reported to date have observed simultaneous BDNF and cortisol change after contemplative interventions. Increased peripheral BDNF and lower serum cortisol was found after 3 months of yoga in patients with depression (*N* = 32) (96). Moreover, increased BDNF, but greater CAR, was observed after a 3-month combined yoga and meditation training intervention (*N* = 38) (97). Our study provides evidence that BDNF increase is associated with simultaneous and comprehensive cortisol reduction after purely mental training, unconfounded by effects of physical activity (95), and further reveals HCC reduction as a mediating pathway. The estimated 15.1% mediation is sizable, especially considering that HCC and BDNF were measured in different modalities, which can confound covariance (67). Nonetheless, the partial mediation also indicates that additional processes may contribute to BDNF upregulation after training. For example, cognitive training and related long-term potentiation can increase BDNF levels (2,87,98) and is a common component of contemplative mental training (58,99, 100, 101).

We also found that greater BDNF increase after mental training was linked to individually greater DGV increase. Hippocampal morphology overall is known to be affected by both the neurotoxic effects of chronic stress (10,19,102) and BDNF-mediated neuroplasticity (2). The dentate gyrus in particular expresses peak density of TrkB receptors (53) and is a potential site of BDNF-mediated adult neurogenesis (103), presumably rendering this subfield most sensitive to neuroplastic effects of BDNF [also see (54)]. The DGV association identified here is also consistent with animal work that has shown that chronic stress specifically inhibits BDNF expression in the dentate gyrus (28). Although we previously found no baseline associations between BDNF and dentate gyrus morphology (54), it is conceivable that correspondence emerges only in the more sensitive context of individual responsiveness to training.

While BDNF change was associated specifically with DGV increase, we recently found that the CA1–3 subfield volume was most increased by the training overall, in association with reduced diurnal cortisol output (104). CA volume change may have been mediated by neurotrophins other than BDNF, such as the fibroblast growth factor family (24). Given that we found no main effect of training on DGV, there was no evidence for a mediation pathway from training-related BDNF increase to overall enhanced DGV. Nonetheless, the identified individual difference associations may encourage future investigations of this pathway. More precise measurement methods or larger samples may help detect presumably subtle training effects on brain morphology.

It is noteworthy that BDNF does not have uniformly positive and enhancing effects [see (105, 106, 107) for examples]. Findings from animal studies in particular have demonstrated the complex relationships between BDNF action, stress exposure, and brain structure and function, which appear to depend on stress context (e.g., acute vs. chronic stress), brain region, and type of BDNF (e.g., BDNF, proBDNF, or BDNF messenger RNA) (106) examined. However, for human studies and the less-specific peripheral BDNF measurements, the literature overwhelmingly converges on neuroprotective effects, especially in relation to stress-related disorders (8,15,16,24). Thus, overall, our findings add to the growing literature on positive neurobiological changes following contemplative training (30,37,44,108), including enhanced neuroplasticity (58,104,109), and support the hypothesis that neurotrophins such as BDNF mediate these changes, potentially in conjunction with stress reduction (44,45).

The current work is subject to limitations. We observed an unsystematic and surprisingly strong BDNF change in the passive control cohort for which no fully compelling explanation could be found and which we suggest may be a symptom of measurement error. General test-retest instability and sensitivity of peripheral BDNF assays to state effects and pre-analytical handling (59,110) represent limitations to using BDNF as a biomarker. Here, the reliability of BDNF training effects was corroborated by cortisol mediation and participant-level associations. Nonetheless, our data overall highlight issues with using serum BDNF as an outcome for longitudinal mental training and as an indicator of longer-term neurotrophic action. Future research may take measures to increase the stability of peripheral BDNF measurements, such as pooling multiple samples from closely spaced blood draws (111).

Relatedly, although the ReSource study was designed with a passive control cohort, our analyses focused on simple pre-post intervention comparisons in the combined training cohorts. This approach avoids biasing effect estimates through comparison to the potentially confounded control cohort BDNF, but it also weakens the inferential power of the study. Specifically, it fails to control for potential unknown confounding variables or nonspecific temporal effects, thus limiting causal conclusions about the underlying drivers or active ingredients of the training cohort effects.

While BDNF increased in all training cohorts, this pattern was only marginally significant in TC1 and was nonsignificant in the 3-month TC3 when examined separately (although the BDNF increase in TC3 would presumably have been stronger if training had lasted for more than 3 months). The interpretation of these somewhat heterogeneous results is complicated by the complex design of the ReSource Project, which investigated not only long-term training effects compared to a passive control cohort but was also specifically developed to compare potentially differential module effects within the training cohorts. Future randomized trials may choose a simpler between-groups design to allow a more powerful comparison of simple long-term training effects compared with natural longitudinal BDNF variation.

Although we used a validated and accurate segmentation algorithm to derive HCV and DGV (73), automatic segmentations of standard-resolution T1 images are never perfect, and presumably subtle HCV changes are difficult to capture in vivo. Here, we applied intensive manual controls to support segmentation accuracy. While our results are conceptually consistent with findings from animal and patient studies, the association between training-related BDNF and DGV change requires direct replication in healthy human adults.

### Conclusions

Stress-related downregulation of BDNF has been linked to neurodegenerative processes and has been proposed as a key mediator in the etiology of mood disorders such as depression. Here, we found that 9-month training in attention-based mindfulness, socio-cognitive, and socio-affective practices that reduce stress can boost BDNF levels of healthy adults. We identified reduced long-term HPA axis activity and cortisol exposure as a pathway through which mindfulness-based practices exert their beneficial effects on BDNF availability.

By reducing psycho-endocrine stress load in the general populations, contemplative mental training may stimulate endogenous BDNF levels, thus promoting brain health and potentially counteracting risk factors for brain disorders. However, single serum BDNF samples appear to be unreliable measures of long-term training neurotrophic effects in healthy adults. Future studies should investigate nonspecific BDNF measurement effects and replicate findings before application in preventive health care.

## Supplementary Material

Document S1 Supplemental Methods, Results, Figures S1–S6, and Tables S1–S10

Key Resources Table
