# Supplementary material for: Serum BDNF Increase After 9-Month Contemplative Mental Training Is Associated With Decreased Cortisol Secretion and Increased Dentate Gyrus Volume: Evidence From a Randomized Clinical Trial
Source: Biol Psychiatry Glob Open Sci. 2024 Nov 10;5(2):100414. doi: 10.1016/j.bpsgos.2024.100414 (PMC11786774; doi:10.1016/j.bpsgos.2024.100414)
Supplement: Document S1 — Supplemental Methods, Results, Figures S1–S6, and Tables S1–S10 [file mmc1.pdf]

SUPPLEMENTARY INFORMATION

Serum Brain-Derived Neurotrophic Factor Increase After 9-Month Contemplative Mental Training Is Associated With Decreased Cortisol Secretion and Increased Dentate Gyrus Volume

Puhlmann *et al.*

Table of Contents

Supplementary Methods 2

Supplementary Results A. 7

Figure S1. Baseline associations. 7

Table S1. Sample N per timepoint and cohort 8

Table S2. Raw serum BDNF data per timepoint and cohort 9

Table S3. ANOVA pairwise contrasts of Cohorts serum BDNF levels at study baseline (T0) 10

Table S4. Follow-up contrasts between Cohorts (Training Cohorts versus RCC) 11

Table S5. Follow-up contrasts within Cohorts 12

Supplementary Results B. Role of seasonal variables in BDNF change 13

Figure S2. Associations of BDNF levels with seasonal variables 13

Table S6. Follow-up contrasts within LMM controlling for average sun hours in Berlin and Leipzig 14

Table S7. Effect of training on BDNF and HCC in combined training cohorts 15

Table S8. Longitudinal associations of simple scores in the TCs 16

Figure S3. Results of Mediation analysis with timepoint as a categorical variable (T0-T3) 17

Table S9. Causal Mediation results per timepoint 17

Figure S4. Density plots of cumulative BDNF change 18

Figure S5. BDNF-HCV associations by subfield and hemisphere 19

Figure S6. Subject-level change associations in the retest control cohort 20

Table S10. Associations of changes scores in the Training Cohorts 21

Table S11. Associations of change scores in the Retest Control Cohort 22

Supplementary References 23

Supplementary Methods

S2.1 Participants

As part of the ReSource Project (1), 362 meditation-naïve, healthy adults were recruited from the general public using flyers, advertisements, TV and radio announcements. Volunteers were screened for Axis-I disorder within the past 2 years, and lifetime incidence of schizophrenia, psychotic disorder, bipolar disorder, substance dependency or any Axis-II disorders, using the SCID-I, DIA-X and the SKID-II interview with a trained clinical psychologist (58,60). All participants were meditation naïve. Following dropout before study commencement, the initial sample consisted of N=332 participants (175 women; mean age [SD]: 40.5 [9.3] years) distributed across a passive retest control cohort (RCC, n=90) and three training cohorts (TC1, n=80; TC2, n=81; TC3, n=81) (Figure 1A of the main manuscript). Participants were randomly assigned to cohorts using bootstrapping without replacement to ensure demographically homogeneous groups. Participants gave written informed consent, could withdraw from the study at any time, and were financially compensated. The study was conducted in compliance with the Declaration of Helsinki and approved by the research ethics committees of the University of Leipzig (ethic number: 376/12-ff) and Humboldt University in Berlin (ethic numbers: 2013-20, 2013-29, 2014-10), Germany. Extensive detail on participants and recruitment is reported in ((4)).

S2.4 Measures

S2.4.1 Brain-derived neurotrophic factor (BDNF).

Measurement of BDNF concentration was added as an outcome measure to the ReSource Project subsequent to the trial registration in 2013, in light of increasing interest in its interaction with training-related stress reduction and potential mediating role in neurocognitive changes (5). Peripheral BDNF levels were determined in serum, which is a more reliable marker than plasma BDNF (6). To determine serum levels of BDNF, 5.5 ml of blood was collected into serum vacutainers (Sarstedt, Nümbrecht, Germany). Each participant was asked to provide their samples at the same time of day throughout the study (mean deviation in sampling time [SD]: −0.087 [2.30] hrs) to control for diurnal fluctuations. Blood was allowed to clot for 30 to 45 min and subsequently centrifuged at 3500 rpm for 15 min. Serum was frozen at −80°C until assay. BDNF concentrations in serum were determined at the Department of Clinical Biochemistry, "Aghia Sophia" Children's Hospital, Athens, Greece with a quantitative sandwich enzyme immunoassay technique (R&D Systems, Inc. Minneapolis, MN, USA), using the recommended buffers, diluents and substrates. Optical density of the colour reaction was read using a microtiter plate reader set at 450 nm. BDNF concentrations (in pg/ml) in each sample were calculated according to a standard curve. According to the manufacturer, the minimum detectable dose of total BDNF ranged from 0.372-1.35 pg/mL, with a mean value of 0.997 pg/mL. The intra- and inter-

assay coefficients of variation of <7% were determined by duplicate analysis of > 6% of randomly selected samples. See also (7).

S2.4.2 Cortisol measures

Long-term systemic cortisol release. Hormone concentrations (Hair cortisol [HCC] and cortisone [HEC] concentration) in proximal 3 cm segments of hair were analyzed to assess accumulation of cortisol over 3-month periods (Wennig, 2000), using liquid chromatography-tandem mass spectrometry (LC–MS/MS), the current gold-standard approach for hair steroid analysis (8), and following a previously published protocol with a limit of quantification for cortisol and cortisone below 0.09 pg/mg and intra- and inter-assay CVs between 3.7 and 8.8% (9).

HCC and HEC indicate systemic cortisol exposure and chronic stress (10). Cortisone is an inactive metabolite and precursor molecule to cortisol, and it has been suggested that it yields a complementary, potentially more stable estimate of glucocorticoid exposure than cortisol itself (11). Presumably, HCC and HEC accumulate as free cortisol and cortisone molecules are continuously incorporated into hair follicles during growth, approximately proportional to their systemic levels. HCC and HEC in a 1-cm hair segment should thus reflect the cumulative systemic exposure over an approximately 1-month period (10). See (12) for further details on the assessment of HCC and HEC.

Stress-induced cortisol release. Saliva samples for the measurement of stress-reactive cortisol secretion were collected during an acute laboratory stress challenge, the Trier Social Stress Test (TSST) (13). The TSST is the most frequently used protocol for standardized psychosocial stress induction in the laboratory, and reliably elicits physiological and psychological stress responses (14). Saliva was sampled into Salivette collection devices (Sarstedt, Nümbrecht, Germany), which were stored at −30 °C until assay. For more details on the assessment of acute stress reactivity and prior statistical analysis, see (15,16).

Several indices of acute cortisol release were examined. We previously identified reduced acute cortisol reactivity following the mindfulness-based mental training intervention (15). In line with these analyses, cortisol reactivity was here operationalized as the increase from pre-TSST baseline levels to the timepoint of group-average peak levels (min 20 into the TSST), computed as baseline-corrected peak levels via residualisation (Cinc). Next to cortisol reactivity, the timely downregulation or recovery from a stress response is considered a hallmark of healthy HPA axis functioning (17,18). We also recently found that cortisol reactivity and recovery relate differently to health-related indices (19), as well as to acute BDNF dynamics (16). To examine potentially differential relationships with basal BDNF, we therefore additionally examined three relatively unadulterated one-index measures of acute cortisol dynamics: The minimal (Cmin), maximal (Cmax), and change between minimal and maximal cortisol concentration (Cmaxmin) throughout the testing period. Cmaxmin and Cmin were proposed as optimal

indices of reactivity and recovery in a data driven analysis ((20); see also (19)), and Cmax was included due to its marked numeric change in the training context (15).

Diurnal cortisol release. Saliva for diurnal cortisol measurement was also sampled via Salivette collection devices (Sarstedt, Nümbrecht, Germany). Participants were instructed to avoid any oral intake except water for at least 10 min prior to sampling, and otherwise follow their regular daily routines. To collect saliva, participants were asked to place the collection swabs in their mouth for two minutes while refraining from chewing. Salivettes were initially stored in participants' freezers and once returned to the laboratory at − 30 °C until assay.

Computation of diurnal cortisol indices has been described in detail elsewhere (21–23). Briefly, using initial morning samples, the cortisol awakening response (CAR) was operationalized as a change score from first (baseline) measurement to the 30-minute post-awakening sample. The CAR is considered a unique facet of diurnal cortisol output that represents the necessary physiologic enhancement to deal with the anticipated demands of the upcoming day (24–26). Because participants' sampling times were not electronically monitored, the present data do not fully conform to the CAR assessment consensus guidelines (27), which were published after the conception of the present study. The cortisol diurnal slope (i.e., decline over the course of the day) was operationalized as a change score from first to the final sample of the day at 600 min after awakening. A steeper negative diurnal slope is considered an indicator of dynamic and healthy HPA axis functioning (28). Finally, total diurnal cortisol output was computed as the area under the curve with respect to ground (AUC; (29)) using baseline, 240, 360, 480 and 600 min post-awakening cortisol values. Total diurnal cortisol output is presumed to represent tissue exposure to cortisol across the day (28). Because cortisol levels at awakening reflect unique pre-awakening processes (30) and awakening time confounds cortisol secretion, all three indices of diurnal cortisol secretion were corrected for time of awakening and awakening cortisol levels. All reactive and diurnal salivary cortisol levels (expressed in nmol/l) were determined using a time-resolved fluorescence immunoassay with intra-/interassay variabilities of < 10% / 12% (31) at the Department of Biological and Clinical Psychology, University of Trier, Germany.

Previous studies of the ReSource Project reported main training effects on the assessed cortisol measures (12,15,21) or the relation between BDNF and cortisol stress reactivity and recovery (16). For the present work, we exclusively focused on basal BDNF levels and cortisol samples available in the same participants. Thus, differences in the respective study samples exist due to different overlaps in missing data points for basal BDNF and the respective cortisol measures.

S2.4.3 Hippocampal and dentate gyrus volume

MRI acquisition. T1-weighted images were acquired on a 3T Siemens Verio scanner (Siemens) with a 32-channel head coil, using a three-dimensional (3D) magnetization-prepared rapid gradient-echo (MP-RAGE) sequence (176 sagittal slices; repetition time (TR), 2300 ms; echo time (TE), 2.98ms;

inversion time (TI), 900 ms; flip angle, 7°; field of view (FOV), 240 × 256 mm2; matrix, 240 × 256; 1 × 1 × 1 mm3 voxels). All data was collected using the same Imaging hardware and console software (Syngo B17).

Processing of hippocampal volume (HCV) and dentate gyrus volume (DGV), and quality control. Based on the available high-resolution T1-weighted images, we segmented CA1-3, CA4/DG, and subiculum (SUB) using a patch-based algorithm in every subject (for details see (32)). Hippocampal volumes were estimated based on T1 weighted data that were linearly registered to MNI152, such that intracranial volume was implicitly controlled for. Previous validation studies demonstrated that this algorithm has high accuracy for segmenting hippocampal subfields in T1 images with similar resolution (32), and in detecting hippocampal subfield pathology in patients with epilepsy (33). As segmentation algorithms are never perfect, the automatically derived segmentations were additionally manually quality controlled by two independent raters. R.L. and L.P, following a previously pre-registered procedure. In brief, each segmentation was rated for quality on a scale of 1–10, with points being subtracted depending on the severity of detected flaws. Segmentation with average ratings of 5 and lower qualified for reprocessing with the algorithm, after which segmentations were rated again. Any remaining segmentations with average scores lower than 5 were excluded from analysis. Further details on the processing of this hippocampal volume data have been described elsewhere (7).

S2.5 Statistical analyses

Raw cortisol measures were treated with natural log transformations to remedy skewed distributions (12,15,21). Data points diverging >3 standard deviations (SDs) from the respective sample mean were defined as outliers and winsorized to the respective upper or lower boundary of 3 SD (34). All analyses were conducted in R (version 4.2.0; (35)).

Model equation for main training effects on BDNF.

Fixed effects:

$BDNF_{ij} \sim \beta_0 + \beta_1\, age_i + \beta_2\, sex_i + \beta_{3\text{-}5}\, cohort_i + \beta_{6\text{-}8}\, timepoint_j + \beta_{9\text{-}13}\, cohort_i \times timepoint_j$,

Random effects: $BDNF_{ij} \sim \beta_{0i} + \varepsilon_{ij}$,

where β_0 = intercept, i = subject, j = measurement timepoint (T0, T1, T2, T3), ε = error

Mediation analysis

We next examined whether the group-level BDNF increase after training was mediated by reduced cortisol exposure. Mediation analyses were conducted with the above combined cohort model to avoid multiple comparisons as sample size requirements are generally large for mediations (36). Training effects were again modelled as the linear effect of timepoint after baseline, since effects on BDNF emerged in pre-post comparisons within training cohorts and followed a mostly cumulative pattern.

Two prerequisites for testing any mediation are that a), the independent variable affects the mediating variable (herein: that there is an effect of training on cortisol), *and b*), the mediating variable affects the outcome (herein: that cortisol is associated with BDNF) (37). Based on prerequisite a), we initially identified four potential mediators (i.e., four cortisol indices that we previously found were reduced by the ReSource training (12,15,21), namely HCC and HEC [long-term average cortisol release], Cinc [stress-induced cortisol increase], and CAR [diurnal cortisol awakening response]).

Multilevel analyses of BDNF-cortisol associations showed that among the four potential mediators, only HCC was associated with BDNF levels, in line with prerequisite b) (see "*Simple score associations"* below and Figure 4A, Table S8). Thus, only HCC qualified for mediation analysis. Figure 3 (panel A1, B1) shows the estimated BDNF increase and simultaneous HCC reduction after 3-9 months training (see also Table S7).

We conducted two mediation analyses: First, mediation of the above identified linear effect of timepoint in the combined training cohorts via HCC, and second, moderated mediation analysis of the linear timepoint effect in the training cohorts compared to the control cohort (Training x timepoint in a joined mediation model). In previous work, we already demonstrated that in the RCC there was no effect of the independent variable timepoint on the mediating variable HCC (i.e., HCC in the RCC remained stable over the 9 months observation period (12)). An effect of the independent variable on the mediating variable is a prerequisite for mediation, such that on statistical ground, there should be no mediation of BDNF change via HCC in the RCC.

Supplementary Results A.

Baseline contrasts. Baseline BDNF levels did not differ by sex ($t(313)=0.80$, $p>.25$), hormonal status (woman taking oral contraceptives, woman naturally cycling, woman in menopause, man; $F(2, 303)=2.03$, $p=0.11$, controlled for age) or smoking status (yes/no; $t(292)=0.73$, $p>.25$), but the short 3-month cohort TC3 had lower values than all other study cohorts (omnibus-test: $F(2, 310)=3.84$, $p=.010$; binary contrasts in Table S3).

Figure S1. Baseline associations.

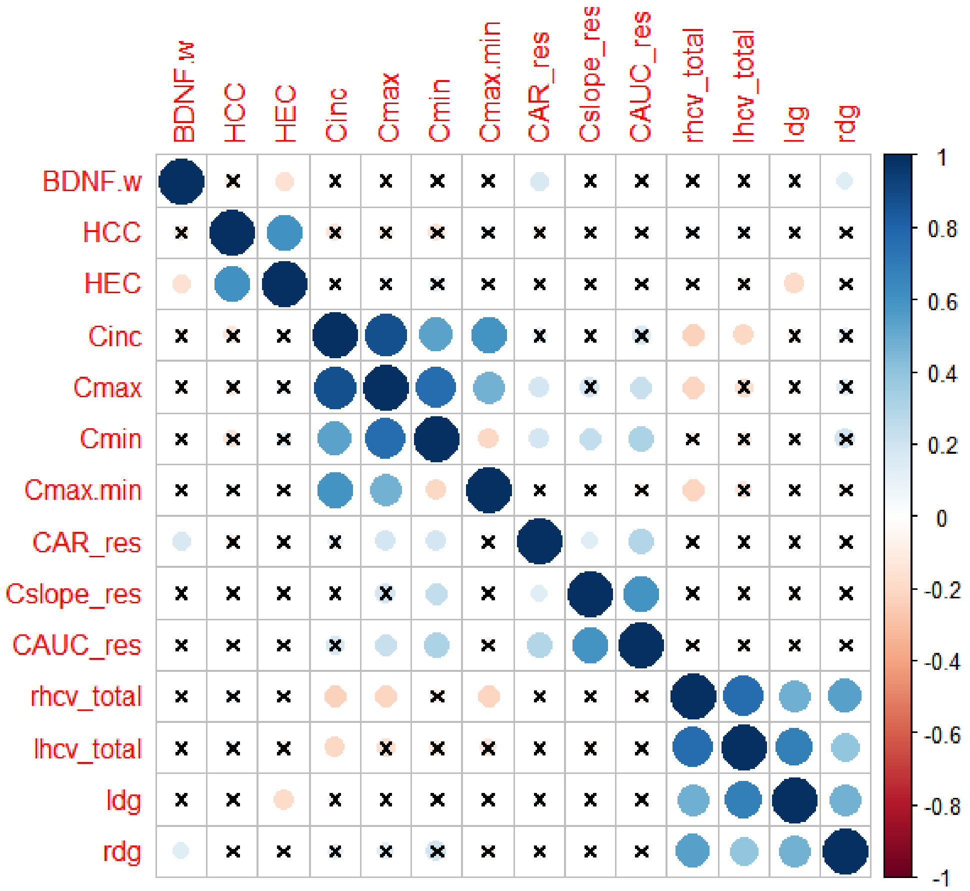

Figure S1. Simple bivariate Pearson correlations between individual difference variables of interest at study baseline (T0) across all cohorts. Note that the association between BDNF and hair cortisol is marginal when controlling for sex and age (beta = -0.15, p = .0502).

Table S1. Sample N per timepoint and cohort

timepoint	Cohort	BDNF	HCC	HEC	Cinc	Cmax	Cmin	Cmaxmin	CAR (raw)	CAR (cor.)	Cslope (raw)	Cslope (cor.)	CAUC (raw)	CAUC (cor.)	HCV	DG
T0	RCC	80	42	50	40	39	39	39	85	85	85	85	84	84	76	76
T0	TC1	75	39	41	7	7	7	7	77	77	76	76	75	75	74	74
T0	TC2	80	38	44	8	8	8	8	79	79	77	77	73	73	67	67
T0	TC3	80	37	42	30	29	29	29	78	77	78	77	77	76	71	71
	**Total**	**315**	**156**	**177**	**85**	**83**	**83**	**83**	**319**	**318**	**316**	**315**	**309**	**308**	**288**	**288**
T1	RCC	74	34	38	19	19	19	19	82	81	78	77	78	77	73	73
T1	TC1	76	27	38	22	22	22	22	75	75	73	73	70	70	67	67
T1	TC2	76	33	37	21	21	21	21	78	78	77	77	73	73	64	64
T1	TC3	72	36	42	45	43	43	43	74	72	73	71	69	67	68	68
	**Total**	**298**	**130**	**155**	**107**	**105**	**105**	**105**	**309**	**306**	**301**	**298**	**290**	**287**	**272**	**272**
T2	RCC	69	50	58	18	18	18	18	83	80	81	78	76	73	67	67
T2	TC1	72	29	37	44	43	43	43	75	74	73	72	71	71	62	62
T2	TC2	72	33	36	44	44	44	44	76	76	73	73	72	72	64	64
	**Total**	**213**	**112**	**131**	**106**	**105**	**105**	**105**	**234**	**230**	**227**	**223**	**219**	**216**	**193**	**193**
T3	RCC	74	43	51	0	0	0	0	79	75	73	70	70	67	68	68
T3	TC1	69	29	39	0	0	0	0	72	69	70	67	67	64	59	59
T3	TC2	73	52	56	0	0	0	0	75	74	73	72	71	70	63	63
	**Total**	**216**	**124**	**146**	**0**	**0**	**0**	**0**	**226**	**218**	**216**	**209**	**208**	**201**	**190**	**190**

Note. Due to missing data in cortisol awakening time and awakening samples, the N for corrected diurnal cortisol data (cor.) is slightly lower than for raw data. Detailed descriptions on reasons for missingness have previously been provided in Puhlmann et al., 2021 (hair cortisol and cortisone data), Engert et al., 2017 (acute cortisol reactivity), Engert et al., 2023 (diurnal cortisol data) and Valk et al., 2017 (structural MRI data; comparatively lower N in the present sample is due to exclusions in segmentation quality control, see Supplementary methods and Puhlmann et al., 2021).

Table S2. Raw serum BDNF data per timepoint and cohort

Timepoint	RCC	TC1	TC2	TC3
T0	24916.35 (5359)	25881.48 (5742)	25524.04 (6541)	23019.92 (5779)
T1	23091.22 (5932)	26957.76 (6347)	27540.74 (6097)	23601.90 (5854)
T2	25963.80 (6095)	26471.75 (6457)	28447.18 (6106)	-
T3	26976.46 (6191)	27101.52 (5237)	29166.88 (6348)	-

Note. Mean (SD) raw serum BDNF data in pg/mL per cohort and timepoint.

Table S3. ANOVA pairwise contrasts of Cohorts serum BDNF levels at study baseline (T0)

**contrast**	**estimate**	**SE**	**df**	**t.ratio**	**p.value**
RCC - TC1	-834.21	930.44	309	-0.90	0.37
RCC - TC2	-442.45	915.72	309	-0.48	0.63
**RCC - TC3**	**2.034.30**	**914.77**	**309**	**2.22**	**0.03**
TC1 - TC2	391.76	929.33	309	0.42	0.67
**TC1 - TC3**	**2.868.51**	**929.39**	**309**	**3.09**	**0.00**
**TC2 - TC3**	**2.476.75**	**914.23**	**309**	**2.71**	**0.01**

Table S4. Follow-up contrasts between Cohorts (Training Cohorts versus RCC)

contrast	Cohort	estimate	SE	df	t.ratio	p.value
RCC - TC1	T0	-894,794	932,9229	642,5342	-0,95913	0,337854
RCC - TC2	T0	-528,424	920,1643	636,0155	-0,57427	0,565987
**RCC - TC3**	**T0**	**1924,625**	**920,6992**	**633,4375**	**2,090395**	**0,03698**
TC1 - TC2	T0	366,3701	934,0449	637,664	0,39224	0,695012
TC1 - TC3	T0	2819,419	935,3104	634,3667	3,014421	0,002677
TC2 - TC3	T0	2453,049	922,502	627,6319	2,659126	0,008034
**RCC - TC1**	**T1**	**-3766,06**	**944,3579**	**657,5133**	**-3,98796**	**7,41E-05**
**RCC - TC2**	**T1**	**-4258,06**	**941,9095**	**666,0367**	**-4,52067**	**7,3E-06**
RCC - TC3	T1	-636,833	951,7844	675,8523	-0,66909	0,503664
TC1 - TC2	T1	-492,002	942,1325	644,7345	-0,52222	0,601696
TC1 - TC3	T1	3129,225	952,6657	653,8847	3,284704	0,001075
TC2 - TC3	T1	3621,226	950,3376	662,4462	3,810463	0,000152
RCC - TC1	T2	-271,629	963,4781	688,5975	-0,28193	0,778085
**RCC - TC2**	**T2**	**-1958,26**	**961,2104**	**696,9487**	**-2,03729**	**0,041998**
RCC - TC3	T2	NA	NA	NA	NA	NA
TC1 - TC2	T2	-1686,64	958,8531	671,6513	-1,75901	0,079031
TC1 - TC3	T2	NA	NA	NA	NA	NA
TC2 - TC3	T2	NA	NA	NA	NA	NA
RCC - TC1	T3	-92,6111	958,8482	681,6753	-0,09659	0,923084
**RCC - TC2**	**T3**	**-1902,56**	**947,9517**	**675,746**	**-2,00702**	**0,045145**
RCC - TC3	T3	NA	NA	NA	NA	NA
TC1 - TC2	T3	-1809,95	963,3595	679,1448	-1,87879	0,060702

Note: Estimates derived from follow-up contrasts within main LMM of BDNF by cohort and timepoint. Significant differences relative to study baseline are highlighted in Bold. TC, Training Cohort; RCC, Retest Control Cohort.

Table S5. Follow-up contrasts within Cohorts

contrast	Cohort	estimate	SE	df	t.ratio	p.value
**T0 - T1**	**TC2**	**-2008.434**	**658.078**	**729.226**	**-3.052**	**0.002**
**T0 - T2**	**TC2**	**-2692.457**	**670.202**	**733.759**	**-4.017**	**0.000**
**T0 - T3**	**TC2**	**-3456.504**	**667.225**	**733.092**	**-5.180**	**0.000**
T1 - T2	TC2	-684.023	672.379	718.303	-1.017	0.309
T1 - T3	TC2	-1448.070	669.429	717.548	-2.163	0.031
T2 - T3	TC2	-764.047	676.749	714.250	-1.129	0.259
**T0 - T1**	**TC1**	**-1150.062**	**666.627**	**722.206**	**-1.725**	**0.085**
T0 - T2	TC1	-639.451	678.607	726.502	-0.942	0.346
**T0 - T3**	**TC1**	**-1280.187**	**687.999**	**728.859**	**-1.861**	**0.063**
T1 - T2	TC1	510.611	672.196	717.648	0.760	0.448
T1 - T3	TC1	-130.125	681.483	719.987	-0.191	0.849
T2 - T3	TC1	-640.736	690.190	719.159	-0.928	0.354
T0 - T1	TC3	-840.257	670.275	737.497	-1.254	0.210
**T0 - T1**	**RCC**	**1721.202**	**665.250**	**729.892**	**2.587**	**0.010**
T0 - T2	RCC	-1262.616	681.104	734.772	-1.854	0.064
**T0 - T3**	**RCC**	**-2082.370**	**663.508**	**726.413**	**-3.138**	**0.002**
T1 - T2	RCC	-2983.818	688.439	723.504	-4.334	0.000
T1 - T3	RCC	-3803.572	678.931	728.982	-5.602	0.000
T2 - T3	RCC	-819.754	691.638	728.914	-1.185	0.236

Note: Estimates derived from follow-up contrasts within main LMM of BDNF by cohort and timepoint. Significant differences relative to study baseline are highlighted in Bold. TC, Training Cohort; RCC, Retest Control Cohort.

<u>Supplementary Results B.</u> Role of seasonal variables in BDNF change

BDNF levels in longitudinal analyses can also be confounded by seasonal changes, in particular ambient sunlight (38). We explored this potential confound by examining associations between BDNF and the average number of sunlight hours, as well as light hours and temperature in the month preceding each blood sampling. Seasonal weather data was taken from the German weather service (Deutscher Wetterdienst, https://www.dwd.de), separately for the two sites of recruitment, Berlin and Leipzig, Germany.

Figure S2. Associations of BDNF levels with seasonal variables

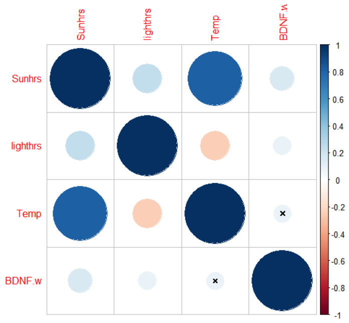

	Sunhrs	lighthrs	Temp
Sunhrs	1.000	-	-
lighthrs	0.247*	1.000	-
Temp	0.817*	-0.244*	1.000
BDNF.w	0.165*	0.095*	0.090

Figure S2. Baseline correlations between BDNF concentration and average hours of sunlight, daylight, and temperature at the site of data collection in the month preceding measurement. Table: Pearson r of bivariate correlations. Asterisks indicate significant correlation at $p < .05$.

At baseline, weather data was positively correlated except for temperature and daylight hours (Figure S3). Baseline BDNF correlated most strongly and positively with average hours of sunlight (Pearson r=.16, p = .003, df=311). Controlling for sunlight in the main LMM of training effects did, however, not significantly increase the explained variance (p>.25) and there was not associated with BDNF in the longitudinal model. The overall pattern of results also remained the same (except for rendering change in TC1 significant and TC3 marginal, see Table S3).

Table S6. Follow-up contrasts within LMM controlling for average sun hours in Berlin and Leipzig

ontrast	Cohort	estimate	SE	df	t.ratio	p.value
**T0 - T1**	**TC2**	**-3,041.630**	**1,010.497**	**698.609**	**-3.010**	**0.003**
**T0 - T2**	**TC2**	**-3,314.588**	**818.502**	**679.485**	**-4.050**	**0.000**
**T0 - T3**	**TC2**	**-3,728.284**	**987.706**	**685.459**	**-3.775**	**0.000**
T1 - T2	TC2	-272.958	732.510	651.931	-0.373	0.710
T1 - T3	TC2	-686.654	1,010.001	681.899	-0.680	0.497
T2 - T3	TC2	-413.696	934.129	669.274	-0.443	0.658
**T0 - T1**	**TC1**	**-1,716.644**	**785.340**	**666.924**	**-2.186**	**0.029**
**T0 - T2**	**TC1**	**-1,523.359**	**942.845**	**688.870**	**-1.616**	**0.107**
**T0 - T3**	**TC1**	**-1,542.311**	**740.128**	**656.300**	**-2.084**	**0.038**
T1 - T2	TC1	193.286	710.659	646.177	0.272	0.786
T1 - T3	TC1	174.333	760.536	654.655	0.229	0.819
T2 - T3	TC1	-18.953	877.771	673.443	-0.022	0.983
**T0 - T1**	**TC3**	**-1,602.661**	**873.121**	**685.989**	**-1.836**	**0.067**
**T0 - T1**	**RCC**	**1,734.184**	**687.743**	**659.544**	**2.522**	**0.012**
**T0 - T2**	**RCC**	**-1,846.999**	**911.318**	**707.464**	**-2.027**	**0.043**
**T0 - T3**	**RCC**	**-2,486.128**	**803.630**	**672.905**	**-3.094**	**0.002**
T1 - T2	RCC	-3,581.183	823.104	673.433	-4.351	0.000
T1 - T3	RCC	-4,220.312	781.405	664.440	-5.401	0.000
T2 - T3	RCC	-639.129	859.777	687.874	-0.743	0.458

Note: Results of contrasts of model estimated BDNF concentration by cohort and timepoint, controlled for average sun hours in the month before blood sampling. Significant differences relative to study baseline are highlighted in Bold. TC, Training Cohort; RCC, Retest Control Cohort.

Table S7. Effect of training on BDNF and HCC in combined training cohorts

**BDNF**	**Estimate**	**Std. Error**	**df**	**t value**	**Pr(>|t|)**
(Intercept)	-0.158	0.095	300.534	-1.668	0.096
CohortTC2	0.131	0.122	209.996	1.075	0.283
CohortTC3	-0.407	0.129	267.391	-3.146	0.002
age.z	0.196	0.052	227.204	3.779	0.000
sex.z	-0.147	0.105	229.765	-1.401	0.162
timepointT1	0.215	0.060	516.137	3.573	0.000
timepointT2	0.248	0.072	515.613	3.453	0.001
timepointT3	0.362	0.072	516.185	5.023	0.000
**Cortisol**	**Estimate**	**Std. Error**	**df**	**t value**	**Pr(>|t|)**
(Intercept)	0.282	0.137	180.691	2.055	0.041
age.z	0.043	0.067	124.723	0.649	0.518
sex.z	-0.120	0.149	128.474	-0.806	0.422
CohortTC2	0.201	0.165	123.605	1.221	0.224
CohortTC3	-0.007	0.189	157.668	-0.036	0.971
timepointT1	-0.385	0.108	228.823	-3.567	0.000
timepointT2	-0.804	0.131	221.436	-6.132	0.000
timepointT3	-0.540	0.120	225.669	-4.504	0.000

Note: Results of LMMs for effect of training duration (timepoint) on BDNF and HCC.

Table S8. Longitudinal associations of simple scores in the TCs

pred	dv	estimate	std.error	statistic	p.value	conf.low	conf.high	category
**HCC**	**BDNF**	**-0.112**	**0.049**	**-2.271**	**0.024**	**-0.209**	**-0.015**	**Long-term cortisol exposure**
HEC	BDNF	0.013	0.046	0.280	0.780	-0.077	0.103	Long-term cortisol exposure
CAR	BDNF	0.001	0.031	0.047	0.962	-0.059	0.062	Diurnal cortisol
**Cslope**	**BDNF**	**-0.062**	**0.031**	**-2.028**	**0.043**	**-0.122**	**-0.002**	**Diurnal cortisol**
**CAUC**	**BDNF**	**-0.071**	**0.033**	**-2.134**	**0.033**	**-0.136**	**-0.006**	**Diurnal cortisol**
HCV ~ BDNF	HCV	-0.016	0.024	-0.654	0.513	-0.063	0.032	Brain morphology
DGV ~ BDNF	DGV	0.051	0.032	1.620	0.106	-0.011	0.113	Brain morphology
Cinc	BDNF	0.017	0.076	0.218	0.828	-0.134	0.167	Stress-reactive cortisol
Cmax	BDNF	-0.009	0.068	-0.127	0.899	-0.142	0.125	Stress-reactive cortisol
Cmin	BDNF	0.042	0.067	0.624	0.533	-0.090	0.173	Stress-reactive cortisol
Cmaxmin	BDNF	-0.054	0.067	-0.797	0.427	-0.186	0.079	Stress-reactive cortisol

Note: Results of LMMs for associations between simple BDNF scores and individual difference measures in the TCs across all timepoints. Besides testing change score associations, we also examined associations between simple endogenous BDNF levels and simple cortisol, DGV and HCV scores. Simple score associations were derived from multilevel models fit over data from all timepoints and training participants. HCC, Cslope and CAUC were significantly negatively associated with participants' endogenous BDNF levels. Results are visualised in Figure 4A of the main manuscript. TCs, Training Cohorts; LMMs, linear mixed models.

Figure S3. Results of Mediation analysis with timepoint as a categorical variable (T0-T3)

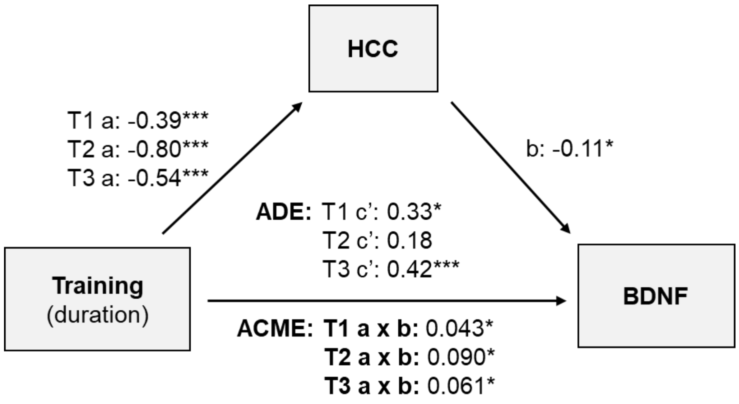

Figure S3. Estimated mediation components in stepwise notation (Baron & Kenny, 1986). Path a: Effect of independent variable 'Training' on mediator HCC; path b: Association between mediator HCC and outcome variable BDNF, estimated across all timepoints of measurement; path c: Total effect of training on BDNF; path a x b: Indirect effect of training via HCC reduction).

Table S9. Causal Mediation results per timepoint

	**Estimate**	**95% CI Lower**	**95% CI Upper**	**p-value**
**T1**				
Total Effect	0.3769	0.1932	0.5641	0.0006
ACME (average)	0.0431	0.0052	0.0948	0.0242
ADE (average)	0.3338	0.1475	0.5208	0.0006
Prop. Mediated (average)	0.1112	0.0136	0.3009	0.0248
**T2**				
Total Effect	0.2722	0.0481	0.4970	0.0182
ACME (average)	0.0897	0.0128	0.1774	0.0230
ADE (average)	0.1825	-0.0547	0.4159	0.1344
Prop. Mediated (average)	0.3223	0.0194	1.5535	0.0408
**T3**				
Total Effect	0.4773	0.2699	0.6836	0.0000
ACME (average)	0.0607	0.0067	0.1265	0.0248
ADE (average)	0.4167	0.2062	0.6282	0.0002
Prop. Mediated (average)	0.1234	0.0139	0.3025	0.0248

Note: Results of Causal Mediation analyses of training effect on BDNF via HCC reduction with timepoint as a categorical variable. ACME, average causal mediation effect; ADE, Average direct effect. HCC

Figure S4. Density plots of cumulative BDNF change

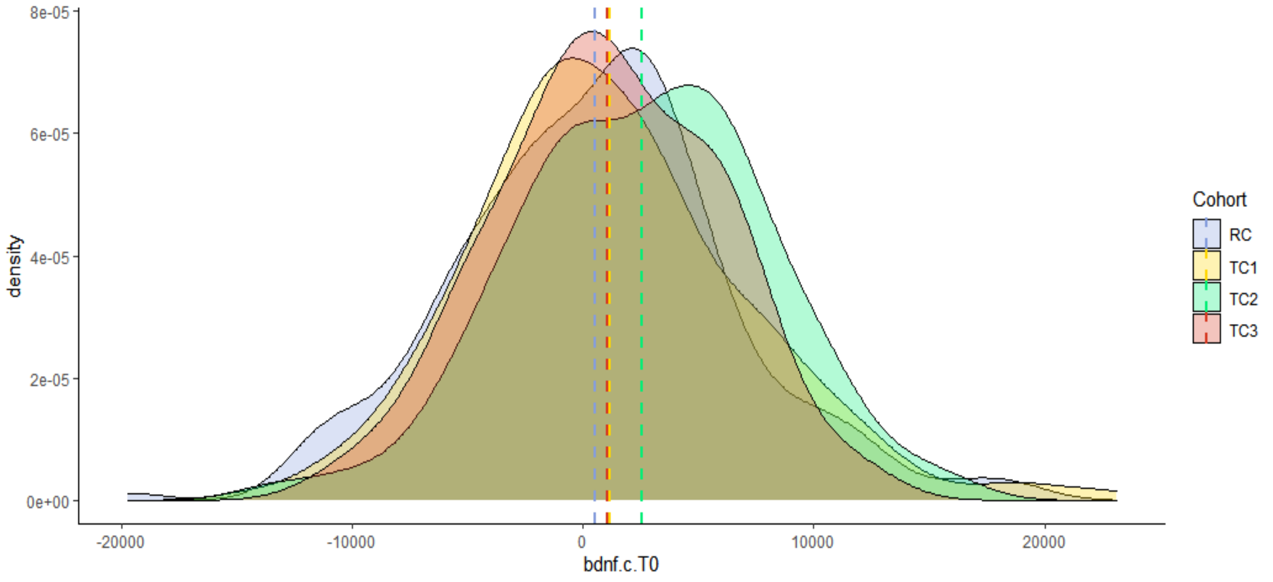

Figure S4. Density plots of cumulative change in serum BDNF levels per cohort across all timepoints. Dashed lines mark cohort means.

Figure S5. BDNF-HCV associations by subfield and hemisphere

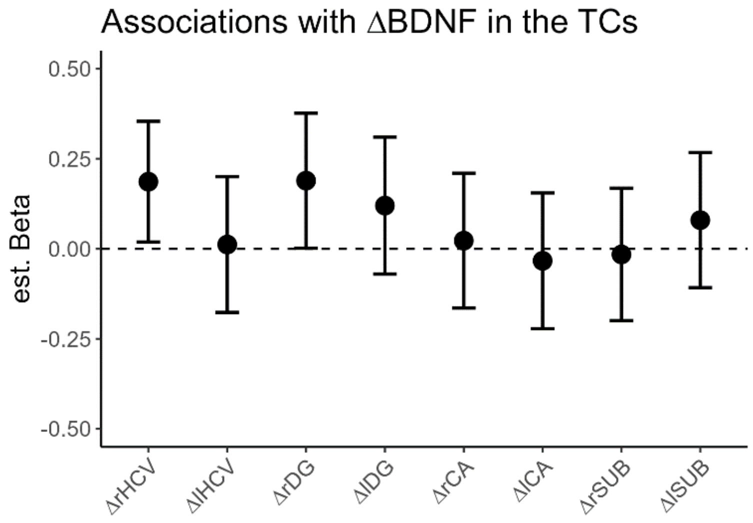

Figure S5. Hippocampal volume to BDNF associations in the TCs by subfield and hemisphere.

Figure S6. Subject-level change associations in the retest control cohort

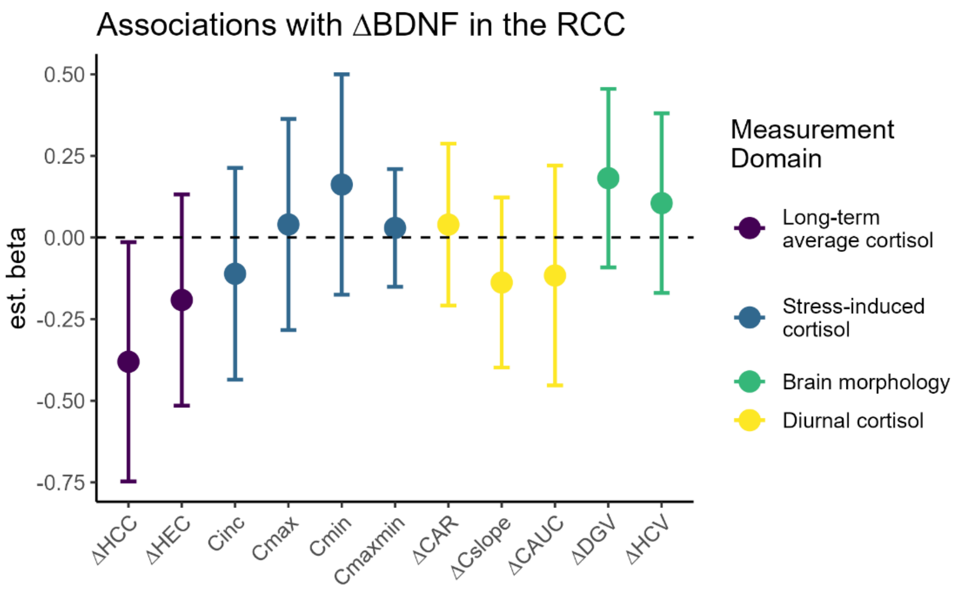

Figure S6. **Associations between subject-level change in BDNF concentration, cortisol measures and hippocampal volume in the control cohort.** Estimated Beta values of associations between changes scores from the pre-training baseline (T0) to the maximum training duration (T3), derived from linear models with the retest control cohort (RCC). For the cross-sectionally sampled stress-reactive HPA axis activity, associations between ΔBDNF and simple cortisol indices (Cinc, Cmin, Cmax, Cmaxmin) were evaluated.

Table S10. Associations of changes scores in the Training Cohorts

	Dependent Variable										
	**ΔBDNF**									**ΔHCV**	**ΔDGV**
ΔHCC	-.28 (-.51;-.04)*										
ΔHEC		-.24 (-.45;-.03)*									
Cinc			-.12 (-.26;.02)°								
Cmax				-.16 (-.30;-.01)*							
Cmin					-.16 (-.30;-.02)*						
Cmaxmin						-.04 (-.19;.10)					
ΔCAR							.03 (-.15;.21)				
ΔCslope								.01 (-.17;.19)			
ΔCAUC									-.11 (-.29;.08)		
ΔBDNF										.03 (-.16;.22)	.19 (.01;.38)*
Constant	.15 (-.07;.36)	.19 (-.02;.39)	-.03 (-.17;.12)	-.03 (-.18;.12)	-.03 (-.18;.11)	-.03 (-.18;.12)	.04 (-.13;.22)	.06 (-.12;.24)	.07 (-.12;.25)	-.001 (-.19;.19)	-.01 (-.20;.17)
Observations	49	64	168	165	165	165	130	125	118	111	110
R^2	0.1	0.07	0.02	0.03	0.03	0.002	0.001	0.0001	0.01	0.001	0.04
Adjusted R^2	0.08	0.06	0.01	0.02	0.02	-0.004	-0.01	-0.01	0.002	-0.01	0.03
Residual Std. Error	.75 (df = 47)	.85 (df = 62)	.96 (df = 166)	.95 (df = 163)	.95 (df = 163)	.97 (df = 163)	1.01 (df = 128)	1.03 (df = 123)	1.03 (df = 116)	1.02 (df = 109)	1.00 (df = 108)
F Statistic	5.42* (df = 1; 47)	4.97* (df = 1; 62)	2.76 (df = 1; 166)	4.55* (df = 1; 163)	4.82* (df = 1; 163)	.31 (df = 1; 163)	.10 (df = 1; 128)	.01 (df = 1; 123)	1.27 (df = 1; 116)	.11 (df = 1; 109)	4.36* (df = 1; 108)

Note: Results of linear models of associations between ΔBDNF scores and change in individual difference measures from baseline to month nine in the TCs. Results are visualised in Figure 4B of the main manuscript. TCs, Training Cohorts. °p<0.1; *p<0.05; **p<0.01; ***p<0.001.

Table S11. Associations of change scores in the Retest Control Cohort

	Dependent Variable										
	**ΔBDNF**									**ΔHCV**	**ΔDGV**
ΔHCC	-.38 (-.73;-.03)*										
ΔHEC		-.19 (-.50;.12)									
Cinc			-.11 (-.42;.20)								
Cmax				.04 (-.27;.35)							
Cmin					.16 (-.16;.49)						
ΔCAR						.03 (-.15;.21)					
ΔCslope							.04 (-.20;.28)				
ΔCAUC								-.14 (-.39;.12)			
Cmaxmin									-.12 (-.44;.21)		
ΔBDNF										-.07 (-.34;.20)	.18 (-.09;.45)
Constant	.13 (-.21;.46)	.07 (-.25;.39)	-.21 (-.53;.10)	-.21 (-.53;.11)	-.21 (-.52;.11)	.04 (-.13;.22)	-.04 (-.29;.21)	-.03 (-.29;.23)	-.21 (-.53;.10)	-.02 (-.28;.24)	-.01 (-.26;.25)
Observations	26	35	34	34	34	130	64	60	34	60	58
R^2	0.16	0.04	0.01	0.002	0.03	0.001	0.002	0.02	0.02	0.005	0.03
Adjusted R^2	0.13	0.01	-0.02	-0.03	-0.001	-0.01	-0.01	0.002	-0.02	-0.01	0.01
Residual Std. Error	.88 (df = 24)	.97 (df = 33)	.94 (df = 32)	.94 (df = 32)	.93 (df = 32)	1.01 (df = 128)	1.01 (df = 62)	1.02 (df = 58)	.94 (df = 32)	1.01 (df = 58)	.98 (df = 56)
F Statistic	4.60* (df = 1; 24)	1.45 (df = 1; 33)	.49 (df = 1; 32)	.06 (df = 1; 32)	.96 (df = 1; 32)	.10 (df = 1; 128)	.10 (df = 1; 62)	1.12 (df = 1; 58)	.50 (df = 1; 32)	.27 (df = 1; 58)	1.77 (df = 1; 56)

Note: Results of linear models of associations between ΔBDNF scores and change in individual difference measures from baseline to month nine in the RCC. Results are visualised in Figure S5. °p<0.1; *p<0.05; **p<0.01; ***p<0.001; 95% CI in brackets. RCC, Retest Control Cohort

Supplementary References

1. Singer T, Kok BE, Bornemann B, Zurborg S, Bolz M, Bochow CA (2016): *The ReSource Project. Background, Design, Samples, and Measurements*, 2nd ed. Leipzig, Germany: Max Planck Institute for Human Cognitive and Brain Sciences.

2. Wittchen, H. U., Zaudig, M., & Fydrich T (1997): *SKID – Strukturiertes Klinisches Interview Für DSM-IV. Achse I Und II*. Göttingen: Hogrefe.

3. Gibbon, M., Spitzer, R. L., & First MB (1997): User's guide for the structured clinical interview for DSM-IV axis II personality disorders: SCID-II.

4. Singer T, Kok BE, Bornemann B, Zurborg S (2016): Recruitment, Sample Description, and Dropout. *The ReSource Project. Background, Design, Samples, and Measurements*, 2nd ed. Leipzig, Germany, pp 44–51.

5. Tang YY, Hölzel BK, Posner MI (2015): The neuroscience of mindfulness meditation. *Nat Rev Neurosci* 16: 213–225.

6. Polyakova M, Schlögl H, Sacher J, Schmidt-Kassow M, Kaiser J, Stumvoll M, *et al.* (2017): Stability of bdnf in human samples stored up to 6 months and correlations of serum and edta-plasma concentrations. *Int J Mol Sci*. https://doi.org/10.3390/ijms18061189

7. Puhlmann LMC, Linz R, Valk SL, Vrticka P, Vos de Wael R, Bernasconi A, *et al.* (2021): Association between hippocampal structure and serum Brain-Derived Neurotrophic Factor (BDNF) in healthy adults: A registered report. *Neuroimage* 236: 118011.

8. Gao W, Kirschbaum C, Grass J, Stalder T (2016): LC–MS based analysis of endogenous steroid hormones in human hair. *Journal of Steroid Biochemistry and Molecular Biology* 162: 92–99.

9. Gao W, Stalder T, Foley P, Rauh M, Deng H, Kirschbaum C (2013): Quantitative analysis of steroid hormones in human hair using a column-switching LC-APCI-MS/MS assay. *J Chromatogr B Analyt Technol Biomed Life Sci* 928: 1–8.

10. Stalder T, Steudte-Schmiedgen S, Alexander N, Klucken T, Vater A, Wichmann S, *et al.* (2017): Stress-related and basic determinants of hair cortisol in humans: A meta-analysis. *Psychoneuroendocrinology* 77: 261–274.

11. Stalder T, Kirschbaum C, Alexander N, Bornstein SR, Gao W, Miller R, *et al.* (2013): Cortisol in hair and the metabolic syndrome. *Journal of Clinical Endocrinology and Metabolism* 98: 2573–2580.

12. Puhlmann LMC, Vrtička P, Linz R, Stalder T, Kirschbaum C, Engert V, Singer T (2021): Contemplative Mental Training Reduces Hair Glucocorticoid Levels in a Randomized Clinical Trial. *Psychosom Med* 83: 894–905.

13. Kirschbaum C, Pirke K-M, Hellhammer DH (1993): The "Trier Social Stress Test" - A Tool for Investigating Psychobiological Stress Responses in a Laboratory Setting. *Neuropsychobiology* 28: 76–81.

14. Allen AP, Kennedy PJ, Cryan JF, Dinan TG, Clarke G (2014): Biological and psychological markers of stress in humans: Focus on the Trier Social Stress Test. *Neurosci Biobehav Rev* 38: 94–124.

15. Engert V, Kok BE, Papassotiriou I, Chrousos GP, Singer T (2017): Specific reduction in cortisol stress reactivity after social but not attention-based mental training. *Sci Adv* 3: 1–13.

16. Linz R, Puhlmann LMC, Apostolakou F, Mantzou E, Papassotiriou I, Chrousos GP, *et al.* (2019): Acute psychosocial stress increases serum BDNF levels: an antagonistic relation to cortisol but no group differences after mental training. *Neuropsychopharmacology* 44: 1797-1804.

17. McEwen BS (1998): Protective and damaging effects of stress mediators. *New England Journal of Medicine* 338: 171–179.

18. McEwen BS (2006): Protective and damaging effects of stress mediators: Central role of the brain. *Dialogues Clin Neurosci* 8: 367–381.

19. Degering M, Linz R, Puhlmann LMC, Singer T, Engert V (2023): Revisiting the stress recovery hypothesis: Differential associations of cortisol stress reactivity and recovery after acute psychosocial stress with markers of long-term stress and health. *Brain Behav Immun Health* 28: 100598.

20. Miller R, Wojtyniak JG, Weckesser LJ, Alexander NC, Engert V, Lehr T (2018): How to disentangle psychobiological stress reactivity and recovery: A comparison of model-based and non-compartmental analyses of cortisol concentrations. *Psychoneuroendocrinology* 90: 194–210.

21. Engert V, Hoehne K, Singer T (2023): Specific Reduction in the Cortisol Awakening Response after Socio-Affective Mental Training. *Mindfulness (N Y)* 681–694.

22. Linz R, Puhlmann LMC, Engert V, Singer T (2022): Investigating the impact of distinct contemplative mental trainings on daily life stress, thoughts and affect—Evidence from a nine-month longitudinal ecological momentary assessment study. *Psychoneuroendocrinology* 142: 105800.

23. Engert V, Kok BE, Puhlmann LMC, Stalder T, Kirschbaum C, Apostolakou F, *et al.* (2018): Exploring the multidimensional complex systems structure of the stress response and its relation to health and sleep outcomes. *Brain Behav Immun* 73: 390–402.

24. Adam EK, Hawkley LC, Kudielka BM, Cacioppo JT (2006): Day-to-day dynamics of experience-cortisol associations in a population-based sample of older adults. *Proc Natl Acad Sci U S A* 103: 17058–17063.

25. Kunz-Ebrecht SR, Kirschbaum C, Marmot M, Steptoe A (2004): Differences in cortisol awakening response on work days and weekends in women and men from the Whitehall II cohort. *Psychoneuroendocrinology* 29: 516–528.

26. Schlotz W, Hellhammer J, Schulz P, Stone AA (2004): Perceived Work Overload and Chronic Worrying Predict Weekend-Weekday Differences in the Cortisol Awakening Response. *Psychosom Med* 66: 207–214.

27. Stalder T, Kirschbaum C, Kudielka BM, Adam EK, Pruessner JC, Wüst S, *et al.* (2016, January 1): Assessment of the cortisol awakening response: Expert consensus guidelines. *Psychoneuroendocrinology*, vol. 63. Elsevier Ltd, pp 414–432.

28. Ross KM, Murphy MLM, Adam EK, Chen E, Miller GE (2014): How stable are diurnal cortisol activity indices in healthy individuals? Evidence from three multi-wave studies. *Psychoneuroendocrinology* 39: 184–193.

29. Pruessner JC, Kirschbaum C, Meinlschmid G, Hellhammer DH (2003): Two formulas for computation of the area under the curve represent measures of total hormone concentration versus time-dependent change. *Psychoneuroendocrinology* 28: 916–931.

30. Clow A, Hucklebridge F, Thorn L (2010): The cortisol awakening response in context. *International Review of Neurobiology*, vol. 93. Academic Press Inc., pp 153–175.

31. Dressendörfer RA, Kirschbaum C, Rohde W, Stahl F, Strasburger CJ (1992): Synthesis of a cortisol-biotin conjugate and evaluation as a tracer in an immunoassay for salivary cortisol measurement. *Journal of Steroid Biochemistry and Molecular Biology* 43: 683–692.

32. Caldairou B, Bernhardt BC, Kulaga-Yoskovitz J, Kim H, Bernasconi N, Bernasconi A (2016): A Surface Patch-Based Segmentation Method for Hippocampal Subfields. *Medical Image Computing and Computer-Assisted Intervention – MICCAI 2016*. Springer, pp 379–387.

33. Goubran M, Bernhardt BC, Cantor-Rivera D, Lau JC, Blinston C, Hammond RR, *et al.* (2016): In vivo MRI signatures of hippocampal subfield pathology in intractable epilepsy. *Hum Brain Mapp* 37: 1103–1119.

34. Dixon WJ (1960): Simplified Estimation from Censored Normal Samples. *The Annals of Mathematical Statistics* 31: 385–391.

35. Team RC (2022): R: A language and environment for statistical computing. Vienna, Austria: R Foundation for Statistical Computing. Retrieved from https://www.r-project.org/

36. Fritz MS, Mackinnon DP (2010): Power analysis for mediation studies. *Psychol Sci* 18: 233–239.

37. MacKinnon DP, Fairchild AJ, Fritz MS (2007): Mediation analysis. *Annu Rev Psychol* 58: 593–614.

38. Molendijk ML, Haffmans JPM, Bus BAA, Spinhoven P, Penninx BWJH, Prickaerts J, *et al.* (2012): Serum BDNF Concentrations Show Strong Seasonal Variation and Correlations with the Amount of Ambient Sunlight. *PLoS One* 7: 1–7.
